# Machine learning prediction of 1-year mortality in older patients with heart failure: a nationwide, multicenter, prospective cohort study

**DOI:** 10.1016/j.lanwpc.2026.101808

**Published:** 2026-02-03

**Authors:** Kanji Yamada, Nobuyuki Kagiyama, Tomoyuki Morisawa, Masakazu Saitoh, Kentaro Iwata, Michitaka Kato, Koji Sakurada, Yuji Kono, Yuki Iida, Masanobu Taya, Yoshinari Funami, Kentaro Kamiya, Tetsuya Takahashi

**Affiliations:** a Department of Physical Therapy, Faculty of Health Science, Juntendo University, Tokyo, Japan; b Department of Rehabilitation, Kobe City Medical Center General Hospital, Kobe, Japan; c Department of Cardiovascular Biology and Medicine, Juntendo University Graduate School of Medicine, Tokyo, Japan; d Committee of the J-Proof HF Registry, Japanese Society of Cardiovascular Physical Therapy, Tokyo, Japan

**Keywords:** Heart failure, Aged, Machine learning, Prognosis, Physical function

## Abstract

**Background:**

Risk prediction in older adults with heart failure (HF) often depends on conventional clinical variables and does not incorporate direct measures of physical function. This study aimed to develop and validate a machine learning model that integrates routinely collected functional assessments to predict one-year all-cause mortality.

**Methods:**

We analyzed data from the J-Proof HF Registry, a nationwide prospective cohort encompassing 96 institutions in Japan. Patients aged ≥65 years who were hospitalized for HF and received a prescription for rehabilitation between December 2020 and March 2022 were included. An eXtreme Gradient Boosting (XGBoost) model was developed using 77 candidate predictors, including demographic, clinical, laboratory, echocardiographic, and directly measured functional variables. Validation employed a leave-one-site-out (LOSO) internal–external framework. Discrimination, calibration, and clinical utility were compared with established risk scores. A 20-predictor model was derived using SHAP-based importance and clinical review.

**Findings:**

Among 9700 eligible patients, the median age was 83 years (interquartile range [IQR] 77–88), 4915 (50.7%) were male, and 1601 (16.5%) died within one year. The full XGBoost model achieved an aggregated area under the receiver operating characteristic curve (AUC) of 0.76 (95% confidence interval [CI] 0.75–0.77) across LOSO test sets. The 20-predictor XGBoost model demonstrated similar discrimination (AUC 0.76; 95% CI 0.74–0.77). Both models outperformed the AHEAD and BIOSTAT compact scores. Functional measures at discharge, including Barthel index, the Short Physical Performance Battery, gait speed, and handgrip strength, were among the strongest contributors to model predictions. Reclassification metrics and decision curve analysis indicated greater clinical utility compared with benchmark scores.

**Interpretation:**

A machine learning model incorporating functional assessments predicts one-year mortality in older patients with HF and improves risk stratification beyond established scores. Functional status at discharge is an important prognostic indicator and may inform post-discharge care planning.

**Funding:**

This work was supported by research funding of Japanese Society of Cardiovascular Physical Therapy and 10.13039/501100001691JSPS KAKENHI Grant Number JP25K02969.

**Research in context**

**Evidence before this study**

We conducted a systematic search of prognostic models for heart failure (HF) in older adults. Existing models present several limitations. Traditional statistical scores, such as the Seattle Heart Failure Model (SHFM) and MAGGIC score, often lack objective, performance-based measures of physical function, which are critical determinants of outcomes in geriatric populations, and have shown attenuated performance in Asian cohorts. While many machine learning (ML) models demonstrate higher predictive accuracy, their clinical applicability is often constrained. Many of these studies rely on retrospective administrative claims data or electronic health records, which typically do not capture granular, objectively measured functional data such as gait speed or physical performance scores. Furthermore, their validation is often limited to single-center or retrospective datasets, hindering confidence in their generalizability. Our review identified a clear need for a prognostic tool that integrates objective functional data obtained from a prospective, multicenter cohort and has been rigorously validated for broad clinical use.

**Added value of this study**

This study directly addresses these gaps by developing and validating an ML model using data from a prospective, nationwide cohort of 9700 older patients with HF across 96 institutions in Japan. Unlike models derived from claims data, our study incorporates directly measured, performance-based functional assessments performed by trained therapists, providing a more holistic view of patient vulnerability. The primary added value is the rigorous validation methodology; we employed a leave-one-site-out (LOSO) internal-external framework, which provides a robust assessment of model generalizability across 96 distinct, unseen institutional settings. Our parsimonious 20-variable model demonstrated acceptable and consistent discrimination (macro-mean area under the receiver operating characteristic curve 0.76) and offered superior clinical utility compared to established scores, as confirmed by significant improvements in Net Reclassification Improvement (NRI) and Decision Curve Analysis (DCA).

**Implications of all the available evidence**

The available evidence, strengthened by our findings, indicates that the future of risk stratification in geriatric HF requires the integration of comprehensive functional assessments with advanced analytical methods. Our study provides a robustly validated, parsimonious model that serves as a practical tool to translate this concept into clinical practice. The model effectively stratifies patients into low-, intermediate-, and high-risk groups, enabling targeted allocation of healthcare resources, such as intensified rehabilitation or closer follow-up, as illustrated in our proposed clinical pathway and prototype web-based tool. This work therefore offers not only a clinically relevant prediction tool for an underrepresented older Asian population but also a methodological blueprint for developing and rigorously validating generalizable, and ultimately implementable, AI models for vulnerable patient groups.

## Introduction

Heart failure (HF) represents a global health challenge, characterized by high morbidity, mortality, and burden on healthcare systems.^1^ In rapidly aging societies, particularly within East Asia such as Japan, the clinical landscape of HF is particularly complex.^2^ Older patients with HF often present with atypical symptoms and are characterized by a high prevalence of comorbidities such as frailty, sarcopenia, and cognitive impairment, leading to heterogeneous clinical outcomes.^3^^,^^4^ These features increasingly position HF as a prototypical geriatric syndrome, where prognosis is influenced not only by cardiac function but also by a complex interplay of physical, cognitive, and social factors.^5^ Consequently, traditional prognostic assessments relying solely on cardiac-specific metrics are often insufficient for this population, highlighting the need for a more holistic approach to risk stratification. Although cardiac rehabilitation (CR) is a key component of comprehensive HF care,^6^^,^^7^ participation remains suboptimal,^8^^,^^9^ underscoring the need for better risk stratification to guide post-discharge planning.

Effective allocation of healthcare resources requires precise risk stratification, yet widely used prognostic models, such as the Seattle Heart Failure Model (SHFM) and the Meta-analysis Global Group in Chronic Heart Failure (MAGGIC) score, have limitations in this context.^10^^,^^11^ These models rely primarily on cardiac-specific and biomedical variables, often underestimating the impact of non-cardiac factors such as physical function, frailty, and nutritional status, which are critical determinants of prognosis in older adults. Furthermore, as these models were developed primarily using data from North American and European populations, their applicability to older adults in Asia, including Japan, remains limited. This tendency to overlook functional and social vulnerabilities can lead to an underestimation of risk in vulnerable patients, limiting their utility in guiding personalized care.

Machine learning (ML) can address these limitations by capturing complex, nonlinear interactions across diverse clinical variables, demonstrating superior predictive performance compared to conventional statistical methods in various cardiovascular contexts.^12^^,^^13^ However, many ML studies rely on retrospective claims data or electronic health records, which do not include crucial information such as objectively measured physical function. Our study addresses these gaps by using a nationwide, multicenter, prospective registry in Japan to integrate performance-based measures of physical function—data easily assessed at the bedside but not captured in claims-based analyses. Therefore, the primary aim of this study is to develop and rigorously validate a clinically practical prognostic model for one-year mortality by integrating conventional clinical variables with these objective functional assessments, thereby enhancing personalized care for this vulnerable population.

## Methods

### Study design and participants

This study is a secondary predictive modeling analysis of The Japanese PT Multi-center Registry of Older Frail Patients With Heart Failure (J-Proof HF) Registry, a prospective, nationwide, multicenter cohort conducted at 96 institutions across Japan.^14^ The registry enrolled participants between December 2020 and March 2022. For this study, we included consecutive patients from the registry aged ≥65 years who were hospitalized for HF and were prescribed physical rehabilitation.

Eligibility criteria included hospitalization for HF, age ≥65 years, and a prescription for physical rehabilitation. Exclusion criteria were: (1) in-hospital death, (2) undergoing invasive procedures during the index hospitalization (e.g., transcatheter aortic valve implantation, MitraClip, or cardiac surgery), (3) acute coronary syndrome as the primary reason for admission, and (4) being bedridden prior to admission.

This study was conducted in accordance with the Declaration of Helsinki. The central Ethics Committee of Juntendo University (Tokyo, Japan; approval number: 19-005) and the ethics committees of all participating institutions approved the protocol. Informed consent was obtained either in writing or via the opt-out method, depending on institutional policy. The study was registered with the University Hospital Medical Information Network (UMIN) Clinical Trials Registry (ID: UMIN000047893). Reporting of predictive models followed the Transparent reporting of a multivariable prediction model for individual prognosis or diagnosis (TRIPOD) and TRIPOD-Artificial Intelligence (TRIPOD-AI) guidelines.^15^

### Data collection

Data for the J-Proof HF registry were collected at admission and discharge.

At admission, demographic data, comorbidities, New York Heart Association (NYHA) functional class, laboratory findings, and echocardiographic parameters were extracted from medical records by research personnel. Pre-hospital functional status, including the Barthel Index (BI)^16^ and Kihon Checklist (KCL) score,^17^ was also documented.

At discharge, licensed physical therapists conducted performance-based functional assessments according to a standardized protocol, including the Short Physical Performance Battery (SPPB),^18^ handgrip strength, gait speed, and limb circumferences. Activities of daily living (ADL) were re-assessed using the BI and, where available, the Functional Independence Measure (FIM).

All data submitted to the central data center underwent a quality control process. Data were first checked for omissions, input errors, and out-of-range values by a dedicated data manager. Queries were resolved by participating institutions, and the cleaned dataset was verified by two investigators before analysis.

### Outcome

The primary outcome was all-cause mortality within one year of hospital discharge. Vital status was ascertained using a hierarchical follow-up protocol: (1) a postal questionnaire, (2) telephone follow-up, and (3) review of medical records for patients who returned to the participating institution. The registry did not include scheduled interim follow-up visits; therefore, survival status was collected only once at the predefined one-year time point. All predictor variables were obtained at the index hospitalization, and no follow-up data were used for model development or validation.

For deceased patients, the date and cause of death were recorded when available and categorized as cardiovascular (CV) or non-CV death. Patients whose vital status could not be confirmed using any of these methods were excluded from the analysis (Supplementary Figure S1).

### Candidate predictors

All available variables in the registry were considered without prior univariable screening. A complete list of candidate predictors, including definitions, measurement methods, and units, is provided in the Supplementary Material (Supplementary Table S1).

Baseline characteristics: age, sex, body mass index (BMI), etiology of HF, NYHA functional class, comorbidities, and history of HF hospitalization. Pre-hospital functional status was assessed using the BI and the KCL.

In-hospital and discharge data: length of stay, discharge disposition, echocardiographic and laboratory findings, and details of physical therapy (e.g., total units, specific training performed such as endurance or resistance training).

Physical and cognitive function at discharge: SPPB, grip strength, gait speed, and limb circumferences (upper arm and lower leg). The SPPB, which assesses balance, gait speed, and lower extremity strength, was administered following the original standardized protocol.^18^ ADL were assessed using the BI and FIM. All assessments were performed by licensed physical therapists. Physical frailty was evaluated using the Japanese Cardiovascular Health Study (J-CHS) Index.^19^ Cognitive impairment was defined as a score below established thresholds on any of the following: Hasegawa Dementia Scale-Revised (HDS-R <21), Mini-Mental State Examination (MMSE <24), Mini-Cognitive Assessment Instrument (Mini-Cog <3), or Montreal Cognitive Assessment-Japanese version (MoCA-J <26).^14^

Medications: Discharge prescriptions were recorded, with particular attention to guideline-directed medical therapies for HF, including angiotensin-converting enzyme inhibitors (ACE-I)/angiotensin receptor blockers (ARB), angiotensin receptor-neprilysin inhibitors (ARNI), beta-blockers, mineralocorticoid receptor antagonists (MRA), and sodium-glucose cotransporter-2 (SGLT2) inhibitors.

### Sample size

The sample size for this study was determined by the number of eligible patients enrolled in the prospective J-Proof HF Registry. Although no formal a priori sample size calculation was performed at the registry's inception, we undertook a post-hoc assessment of the available dataset's adequacy for model development following the criteria of Riley et al.^20^

We performed the calculation for the development of a ML prediction model, assuming a binary outcome with a prevalence of 17.9%, 77 candidate predictor variables, and a conservatively estimated Cox–Snell R^2^ of 0.09. Based on these inputs, the minimum required sample size to achieve a target shrinkage factor of 0.9 and reduce overfitting risk was estimated at 7309 participants, corresponding to 1309 events and approximately 17.0 events per predictor parameter.

Our cohort comprised 9700 participants, exceeding the calculated threshold, which suggests that the dataset is likely sufficient for developing a generalizable ML model with controlled overfitting risk.

### Missing data

The prevalence of missing data for each candidate predictor is detailed in the Supplementary Appendix (Supplementary Table S1). The primary algorithm, eXtreme Gradient Boosting (XGBoost), was selected in part for its inherent ability to handle missing values. For performance-based functional measures such as gait speed, missing data were interpreted as a potentially informative signal of severe functional impairment. No imputation was applied for the primary outcome.

### Statistical analysis

Patient characteristics are summarized as medians with interquartile range (IQR) for continuous variables or as counts with percentages for categorical variables. Standardized mean differences (SMD) were used to compare baseline characteristics by one-year survival status. All statistical analyses were performed using R software (version 4.5.1), and a two-sided p-value <0.05 was considered statistically significant. Further details on the statistical methodology are provided in the Supplementary Appendix.

#### Model development and validation

Our primary predictive models were developed using the XGBoost algorithm,^21^ and their performance was rigorously evaluated using a nested leave-one-site-out (LOSO) cross-validation framework to ensure generalizability across institutions.^22^ In each outer loop, one institution was held out as the test site, while the remaining sites formed the development set. Within the development set, hyperparameters were optimized using five-fold cross-validation with early stopping.

Two XGBoost models were evaluated within this framework: (1) a Full Model utilizing all available predictors, and (2) a parsimonious Top-20 Model derived through a process described under “Predictor selection”. For each LOSO iteration and for both models, a full development pipeline was executed on the training facilities. This involved five-fold cross-validation for hyperparameter tuning, followed by training the final model on all training facilities using the optimized parameters and an early stopping criterion to prevent overfitting.

Model performance was then assessed for each held-out site and aggregated across all 96 iterations to compute macro-mean performance metrics, including the area under the receiver operating characteristic curve (AUC) with 95% confidence intervals (CI) derived via bootstrapping.

To contextualize the performance of our models, we compared our models against two established clinical risk scores: the AHEAD (Atrial fibrillation, Hemoglobin, Elderly, Abnormal renal parameters, Diabetes mellitus) score^23^ and the BIOSTAT (BIOlogy Study to TAilored Treatment in Chronic Heart Failure) compact model.^24^ These were selected due to their development in similar hospitalized HF populations. Other widely cited scores, such as the MAGGIC and SHFM scores, require variables not systematically collected in our registry, precluding a fair comparison. As a further benchmark representing a traditional statistical approach, a Least Absolute Shrinkage and Selection Operator (LASSO) logistic regression model was trained and evaluated using the same LOSO procedure on a complete-case dataset. For the LASSO model, we applied 10-fold cross-validation and selected the penalty parameter based on the minimum cross-validated deviance (lambda).

To ensure that our interpretation reflects the model's performance on unseen data, model interpretability was examined using SHapley Additive exPlanations (SHAP) values computed exclusively on the held-out test set within each fold of the LOSO cross-validation. This approach allows for an unbiased assessment of feature contributions.

#### Predictor selection

The Top-20 XGBoost model was constructed using a hybrid process integrating data-driven and expert-driven feature selection. First, all variables in the Full XGBoost model were ranked according to their mean absolute SHAP values, aggregated from the LOSO analysis. This ranking was reviewed by a consensus panel, consisting of two physical therapists and a cardiologist, who were blinded to model outcomes and selected the final 20 predictors based on a combination of SHAP importance, established clinical evidence, and practical interpretability. The final list of predictors is provided in Box 1.

Box 1 **Predictor variables in the final Top-20 XGBoost model**

The 20 predictor variables for the parsimonious model were selected from the Full XGBoost model based on a combination of their feature importance (mean absolute SHAP value) and a consensus review by two physical therapists and a cardiologist to ensure clinical relevance and non-redundancy. The final variables included in the model are categorized and listed below.

**Demographics and comorbidities (n = 6)**

• Age (years)
• Sex (male)
• History of HF hospitalization
• Medication at discharge: ACE-I/ARB/ARNI
• History of Chronic Obstructive Pulmonary Disease (COPD)
• History of Cancer

**Laboratory and Echocardiographic Data at Admission (n = 7)**

• Serum albumin (g/dL)
• C-reactive protein (log-transformed, mg/dL)
• Serum sodium (mEq/L)
• Estimated Glomerular Filtration Rate (eGFR, mL/min/1.73 m^2^)
• Natriuretic peptide (BNP or NT-proBNP, standardized z-score)
• Left ventricular ejection fraction (LVEF, %)
• Left atrial diameter (LAD, mm)

**Pre-admission and Discharge Functional/Anthropometric Data (n = 7)**

• Kihon Checklist score (points)
• Body Mass Index (BMI) at discharge (kg/m^2^)
• Barthel Index at discharge (points)
• Handgrip strength at discharge (kg)
• Short Physical Performance Battery (SPPB) total score at discharge (points)
• Maximum calf circumference at discharge (cm)
• Maximum gait speed at discharge (m/s)

Note: The variables are grouped by clinical category. A complete data dictionary is available in Supplementary Table S1.

#### Risk stratification and survival analysis

Patients in the full analytical cohort were classified into three risk groups (Low, Intermediate, and High) based on the tertiles of their predicted one-year mortality probabilities. These predicted risks were obtained from the final Top-20 XGBoost model trained on the entire cohort.

Differences in overall survival across the three groups were evaluated using Kaplan–Meier curves, and statistical significance was assessed with the log-rank test.

To further examine the association between predicted risk and the mode of death, the proportions of CV and non-CV deaths were calculated within each risk group among patients who died during follow-up. In addition, the cumulative incidence of CV and non-CV mortality was estimated for each risk group using competing-risks methods, treating the alternative cause of death as a competing event.

#### Assessment of clinical utility

Performance measures were aggregated across the held-out test sets from the nested LOSO cross-validation framework. Sites with fewer than 10 patients or single-class outcomes were excluded.

The model's discriminative ability was assessed using the AUC with 95% CI. We also calculated the area under the precision–recall curve (AUPRC) and used DeLong's test to compare AUC. Model calibration was assessed graphically using calibration plots. Net Reclassification Improvement (NRI) was calculated at a clinically relevant risk threshold of 20% to quantify the improvement in classification performance compared to established benchmark scores (AHEAD score and BIOSTAT compact). Finally, the clinical utility of the parsimonious model was evaluated using Decision Curve Analysis (DCA), which quantifies the net benefit across a range of threshold probabilities.

Since the AHEAD and BIOSTAT compact scores are point-based systems, their raw scores were used as continuous predictors for ROC and DCA analyses. For NRI, the 20% risk threshold was operationalized by applying a percentile-matched cut-point derived from each LOSO training fold. All comparisons against these benchmark scores were performed on the subset of patients for whom all required score components were available (n = 9620), and this cohort was used for all reported comparative performance evaluations to ensure consistency.

#### Model deployment and visualization

To facilitate the clinical application and interpretation of our findings, we developed an interactive web-based application using the R Shiny framework. This application is based on the Top-20 XGBoost model. It provides an intuitive interface for clinicians to input patient data and receive an individualized one-year mortality risk prediction. The tool also visualizes the key drivers of each prediction based on SHAP values and allows for simulating the potential risk reduction achievable through improvements in modifiable functional parameters.

### Role of the funding source

The funders of the study had no role in study design, data analysis, data interpretation, or writing of the report.

## Results

### Baseline characteristics of the study cohort

A total of 10,052 patients were enrolled in the J-Proof HF Registry, a prospective, nationwide, multicenter cohort study, between December 2020 and March 2022 across 96 institutions. The patient disposition is shown in Supplementary Figure S1. After excluding patients who died during hospitalization (n = 335) and those with missing one-year mortality data (n = 17), the final analytical cohort comprised 9700 patients who survived to discharge.

The baseline characteristics of these 9700 patients are summarized in Table 1. The cohort had a median age of 83 years, included 50.7% males, and had a median left ventricular ejection fraction of 49%. Hypertension was common (68.9%), whereas pre-admission functional independence was generally preserved (median BI, 100). During one-year follow-up, 1601 deaths were observed, corresponding to an overall one-year mortality rate of 16.5%.

**Table 1:** Baseline characteristics by 1-year survival status.

Characteristic	Overall n = 9700	Survived at 1 year n = 8099	Died within 1 year n = 1601	SMD
Demographic data				
Age, years	83 [77, 88]	83 [77, 88]	86 [80, 91]	0.439
Male, n (%)	4918 (50.7)	4072 (50.3)	846 (52.8)	0.051
BMI, kg/m^2^	20.7 [18.5, 23.3]	21.0 [18.8, 23.6]	19.6 [17.53, 22.0]	0.404
Prehospital BI, points	100 [85, 100]	100 [90, 100]	90 [70, 100]	0.514
Prehospital KCL, points	11 [7, 14]	10 [7, 14]	13 [10, 16]	0.497
Medical history				
Etiology of HF, n (%)				
Ischemic heart disease	2852 (29.4)	2345 (29.0)	507 (31.7)	0.059
Cardiomyopathic	1037 (10.7)	887 (11.0)	150 (9.4)	0.052
Arrhythmia	4786 (49.3)	4016 (49.6)	770 (48.1)	0.030
Valvular	3694 (38.1)	2991 (36.9)	703 (43.9)	0.142
NYHA, n (%)				0.140
Ⅰ/Ⅱ	2195 (22.6)	1908 (23.6)	287 (17.9)	
Ⅲ/Ⅳ	7481 (77.3)	6170 (76.4)	1311 (82.0)	
History of HF hospitalization, n (%)	3756 (38.7)	2972 (36.6)	784 (49.0)	0.252
Comorbidities, n (%)				
Hypertension	6682 (68.9)	5636 (69.6)	1046 (65.3)	0.091
Diabetes Mellitus	3365 (34.7)	2810 (34.7)	555 (34.7)	0.001
Hyperlipidemia	3050 (31.4)	2590 (32.0)	460 (28.7)	0.071
Chronic Kidney Disease	3941 (40.6)	3150 (38.9)	791 (49.4)	0.213
COPD	718 (7.4)	559 (6.9)	159 (9.9)	0.110
Cancer	1569 (16.2)	1226 (15.1)	343 (21.4)	0.163
Echocardiography				
LVEF, %	49 [34, 61]	49 [34, 61]	49 [35, 62]	0.016
LAD, mm	44 [39, 49]	44 [39, 49]	44 [39, 49]	0.028
E/e’	16.8 [12.2, 22.9]	16.7 [12.2, 22.6]	17.2 [12.5, 24.4]	0.127
Blood tests				
Albumin, g/dL	3.5 [3.2, 3.8]	3.6 [3.3, 3.9]	3.4 [3.0, 3.7]	0.434
eGFR, mL/min/1.73 m^2^	41 [28, 56]	42 [29, 56]	34 [22, 50]	0.315
C-reactive protein, mg/dL	0.49 [0.16, 1.79]	0.45 [0.14, 1.64]	0.77 [0.23, 2.43]	0.132
Hemoglobin, g/dL	11 [10, 13]	12 [10, 13]	11 [10, 12]	0.337
Na, mEq/L	140 [138, 142]	140 [138, 142]	140 [137, 142]	0.148
BNP, pg/mL	554 [309, 989]	538 [297, 958]	648 [386, 1185]	0.178
NT-proBNP, pg/mL	4784 [2245, 10,289]	4504 [2120, 9747]	6495 [3313, 13,159]	0.227
Medications, n (%)				
ACE-I/ARB/ARNI	5330 (55.1)	4595 (56.9)	735 (46.2)	0.215
β-blockers	6373 (65.8)	5400 (66.7)	973 (61.0)	0.120
MRA	3014 (31.1)	2529 (31.3)	485 (30.4)	0.019
SGLT-2 inhibitor	1529 (15.8)	1327 (16.4)	202 (12.7)	0.106
*Functional characteristics*				
Cognitive decline, n (%)	3538 (40.0)	2788 (37.4)	750 (53.6)	0.329
BI at discharge, points	90 [75, 100]	95 [80, 100]	75 [45, 90]	0.703
Grip strength at discharge, kg	18 [13, 24]	18 [14, 25]	15 [10, 20]	0.539
Maximum walking speed, m/s	0.96 [0.70, 1.23]	0.99 [0.73, 1.26]	0.77 [0.57, 1.01]	0.513
Maximum calf circumference, cm	30 [28, 33]	31 [28, 33]	28 [26, 31]	0.571
SPPB, points	7 [3, 11]	8 [3, 11]	4 [0, 8]	0.680

Values are presented as median [interquartile range] or as %.

SMD represents the absolute standardized mean difference between survivors and non-survivors.

Abbreviations: ACE-I, angiotensin-converting enzyme inhibitor; ARB, angiotensin II receptor blocker; ARNI, angiotensin receptor neprilysin inhibitor; BI, Barthel Index; BMI, body mass index; BNP, brain natriuretic peptide; COPD, chronic obstructive pulmonary disease; eGFR, estimated glomerular filtration rate; E/e’, ratio of early mitral inflow velocity to mitral annular early diastolic velocity; HF, heart failure; KCL, Kihon Check List; LAD, left atrial diameter; LVEF, left ventricular ejection fraction; MRA, mineralocorticoid receptor antagonist; Na, sodium; NT-pro BNP, N-terminal pro brain natriuretic peptide; NYHA, New York Heart Association functional classification; SGLT-2, sodium-glucose co-transporter 2; SMD, Standardized Mean Difference; SPPB, Short physical performance battery.

### Model performance and validation

In the primary analysis using LOSO internal–external validation, the Top-20 XGBoost model achieved discrimination comparable to that of the Full model (Table 2). The macro-mean AUCs were 0.76 (95% CI, 0.74–0.77) for the XGBoost model and 0.76 (95% CI, 0.75–0.77) for the Full XGBoost model, with overlapping CI. Reducing the number of predictors from 77 to 20 did not lead to an appreciable loss of performance.

**Table 2:** Performance Summary in each model.

	AUC (95% CI)	AUPRC	Accuracy	Sensitivity	Specificity	PPV	NPV
XGBoost (Full)	0.76 (0.75–0.77)	0.40	0.68	0.72	0.67	0.30	0.92
XGBoost (Top-20)	0.76 (0.74–0.77)	0.39	0.66	0.74	0.64	0.29	0.92
AHEAD score	0.60 (0.58–0.61)	0.21	0.48	0.72	0.43	0.20	0.88
BIOSTAT compact	0.61 (0.59–0.62)	0.22	0.54	0.64	0.52	0.21	0.88

Accuracy, sensitivity, specificity, PPV, and NPV were calculated using the predicted probabilities obtained from the held-out test sets in the LOSO internal–external validation. The optimal probability threshold for each model was determined by maximizing the Youden index. For the AHEAD and BIOSTAT compact scores, the continuous score values were used to generate ROC curves, and threshold-dependent metrics were calculated using the corresponding optimal cut-points. The event rate for one-year mortality in the analytic cohort was 16.5%, which should be considered when interpreting AUPRC values. Performance metrics for all models were computed on a consistent cohort of 9620 patients who had complete data for the benchmark scores and belonged to evaluable sites.

Abbreviations: AUC, Area Under the Receiver Operating Characteristic Curve; AUPRC, Area Under the Precision-Recall Curve; CI, Confidence Interval; LOSO, leave-one-site-out; NPV, Negative Predictive Value; PPV, Positive Predictive Value; XGBoost, eXtreme Gradient Boosting.

**Fig. 1:**
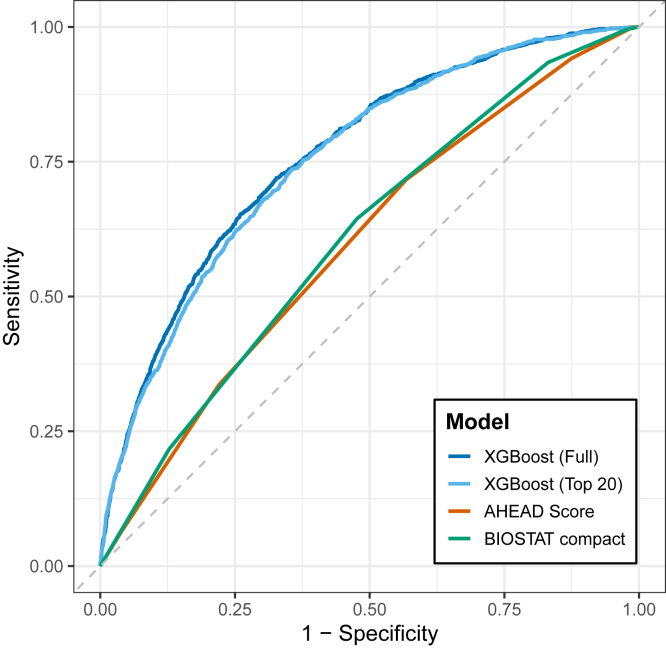
**ROC curve comparison for 1-year mortality prediction**. The figure displays the ROC curves for the Full XGBoost model, the Top-20 XGBoost model, and two established clinical risk scores (AHEAD score and BIOSTAT compact). The dashed diagonal line represents a random classifier with an AUC of 0.50. Abbreviations: ROC, Receiver Operating Characteristic; XGBoost, eXtreme Gradient Boosting.

Both XGBoost models outperformed the AHEAD and BIOSTAT compact scores, which had AUCs of 0.60 (95% CI, 0.58–0.61) and 0.61 (95% CI, 0.59–0.62), respectively (Fig. 1). DeLong's test confirmed that the Top-20 XGBoost model's AUC was significantly higher than both the AHEAD score (absolute difference, 0.16; 95% CI, 0.14–0.18; p < 0.001) and the BIOSTAT compact score (absolute difference, 0.15; 95% CI, 0.13–0.16; p < 0.001). The Full XGBoost model's AUC was statistically superior to the Top-20 XGBoost model's, the absolute difference was minimal (0.007; 95% CI, 0.003–0.011; p = 0.002).

The Top-20 XGBoost model also showed good calibration for the primary outcome, with close agreement between predicted and observed 1-year mortality (Supplementary Figure S2). Applying the model to shorter-term outcomes, such as 30-day and 180-day mortality, resulted in overestimation of risk, as predicted probabilities tended to exceed observed event rates.

### Assessment of clinical utility

To evaluate whether the Top-20 XGBoost model improved patient risk stratification, categorical NRI was calculated at a prespecified 20% risk threshold. Compared with the AHEAD score, the Top-20 XGBoost model achieved an overall NRI of 21.3% (95% CI, 17.8%–24.7%), with positive contributions from both events and non-events. When compared with the BIOSTAT compact score, the overall NRI was 24.0% (95% CI, 20.7%–27.5%), primarily reflecting improved classification of events, whereas classification of non-events showed a modest decline (Supplementary Table S2).

DCA showed that the Top-20 XGBoost model provided greater net benefit than both clinical scores as well as the “treat-all” and “treat-none” strategies across threshold probabilities of 10–40% (Supplementary Figure S3). This pattern was consistent across all LOSO test sets, supporting the potential clinical usefulness of the model.

### Final model performance in the whole cohort

The final prediction model was the parsimonious Top-20 XGBoost model. When trained on the full cohort (n = 9700), it achieved an AUC of 0.81 (95% CI, 0.80–0.82) and an AUPRC of 0.50. Using the Youden index, the optimal probability threshold was 0.18, yielding an accuracy of 0.73, sensitivity of 0.73, specificity of 0.73, a positive predictive value of 0.35, and a negative predictive value of 0.93. The calibration curve demonstrated adequate agreement between predicted and observed risk (Supplementary Figure S4), and DCA indicated a higher net benefit across clinically relevant thresholds compared with existing clinical scores (Supplementary Figure S5).

Model performance was consistent across clinically relevant subgroups, with AUCs ranging from 0.78 to 0.86 (Table 3).

**Table 3:** Performance of the final Top-20 XGBoost model in subgroups.

Subgroup	Number of patients	Number of events (% event rate)	AUC (95% CI)
Overall	9700	1601 (16.5)	0.81 (0.80–0.82)
Age			
<75 years	1730	142 (8.2)	0.86 (0.83–0.89)
75–84 years	3709	524 (14.1)	0.81 (0.79–0.83)
≥85 years	4261	935 (21.9)	0.78 (0.76–0.80)
Sex			
Male	4918	846 (17.2)	0.82 (0.81–0.84)
Female	4782	755 (15.8)	0.80 (0.79–0.82)
LVEF			
HFrEF (LVEF <40%)	3106	484 (15.6)	0.81 (0.79–0.83)
HFmrEF (LVEF 40–49%)	1559	255 (16.4)	0.81 (0.78–0.83)
HFpEF (LVEF ≥ 50%)	4446	717 (16.1)	0.81 (0.79–0.83)
Comorbidity			
With cancer	1569	343 (21.9)	0.81 (0.78–0.83)
Without cancer	8131	1258 (15.5)	0.81 (0.80–0.82)

*Footnote:* Performance was evaluated using final prediction model among subgroups across the entire cohort (n = 9700).

*Abbreviations:* AUC, Area Under the Receiver Operating Characteristic Curve; CI, Confidence Interval; HFrEF, Heart Failure with reduced Ejection Fraction; HFmrEF, Heart Failure with mildly-reduced Ejection Fraction; HFpEF, Heart Failure with preserved Ejection Fraction; LVEF, Left Ventricular Ejection Fraction.

### Model explainability: SHAP analysis

**Fig. 2:**
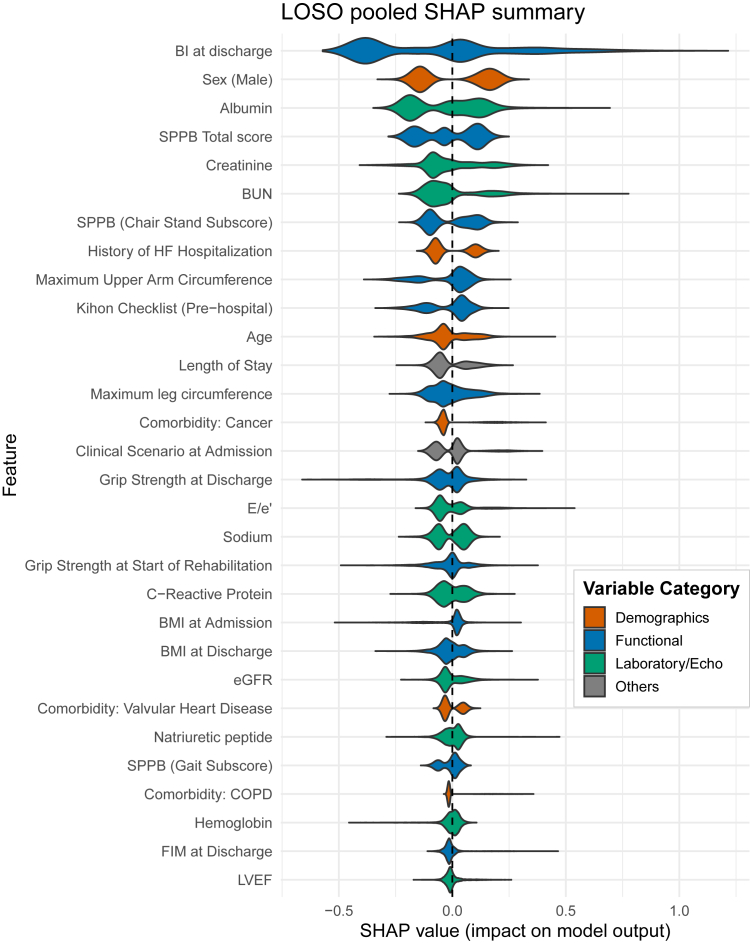
**Feature importance plot for the full XGBoost model**. The plot displays the top 30 predictor variables ranked by their global mean absolute SHAP values. To provide an unbiased evaluation of feature importance on unseen data, SHAP values were calculated solely for patients in the held-out test set of each fold in the LOSO cross-validation. These values were then pooled across all 96 folds to generate the summary shown here. Each violin plot illustrates the distribution of SHAP values for a given feature across out-of-sample predictions, indicating its contribution to the model's estimated one-year mortality risk. Features are ordered from highest to lowest importance. Descriptive variable labels are used for clarity; a complete data dictionary is available in Supplementary Table S1. The color of each feature corresponds to its clinical category, as defined in the legend. Abbreviations: BI, Barthel Index; BMI, Body mass index; BUN, Blood Urea Nitrogen; COPD, Chronic Obstructive Pulmonary disease; E/e’, ratio of early mitral inflow velocity to mitral annular early diastolic velocity; eGFR, estimated Glomerular Filtration Rate; FIM, Functional Independence Measure; HF, Heart Failure; LVEF, Left Ventricular Ejection Fraction; LOSO, leave-one-site-out; SHAP, SHapley Additive exPlanations; SPPB, Short Physical Performance Battery; XGBoost, eXtreme Gradient Boosting.

To characterize the predictors contributing to the Full XGBoost model, a SHAP analysis was performed. The SHAP summary plot (Fig. 2) displays the relative importance and directional effects of the top 30 features. Measures of physical function and frailty—particularly BI at discharge and the SPPB score—were among the most influential predictors. Higher BI scores were associated with lower estimated risk. Serum albumin and sex were also prominent contributors, underscoring the combined relevance of functional status and clinical variables in predicting one-year mortality. Additional visualizations, including a correlation matrix of top predictors (Supplementary Figure S6) and a plot illustrating the feature selection process (Supplementary Figure S7), are provided in the Supplementary Material.

### Risk stratification

**Fig. 3:**
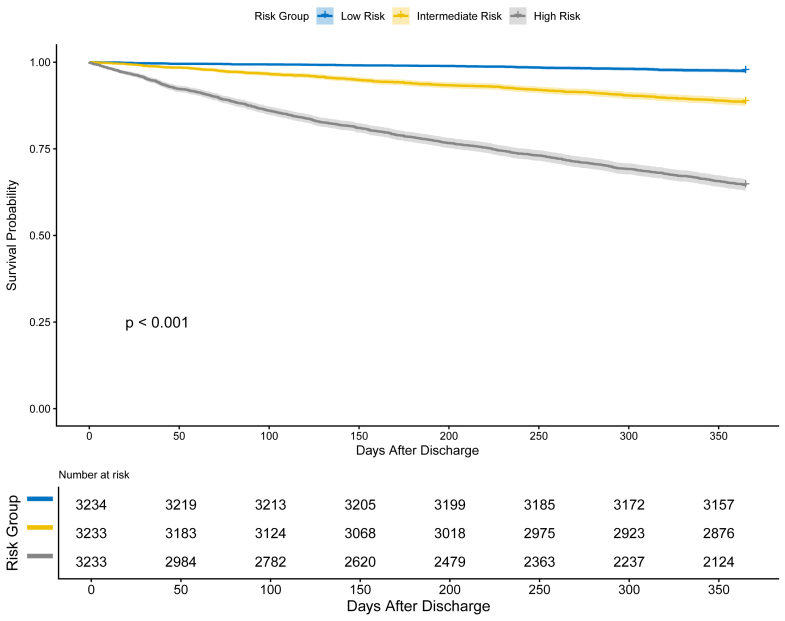
**Kaplan–Meier survival curves for 1-year mortality, stratified by predicted risk**. The figure shows Kaplan–Meier estimates of survival probability, stratified into three risk groups based on the predicted 1-year mortality from the Final Top-20 XGBoost model. Risk groups were defined by tertiles of the predicted probabilities: Low Risk (bottom tertile), Intermediate Risk (middle tertile), and High Risk (top tertile). Shaded areas represent 95% CI. The number of patients at risk in each group is shown below the plot. The p-value was calculated using the log-rank test. Abbreviations: CI, confidence interval; XGBoost, eXtreme Gradient Boosting.

Applying the final Top-20 model to the entire cohort for risk stratification demonstrated clear separation in Kaplan–Meier curves across three risk groups (log-rank p < 0.001; Fig. 3). One-year all-cause mortality rates increased markedly across the risk strata: 2.5% in the Low-risk group, 11.5% in the Intermediate-risk group, and 35.5% in the High-risk group (Supplementary Table S3). A similar gradient was observed for both CV and non-CV mortality. Among the 812 CV deaths, most (n = 719, 88.5%) were attributed to worsening HF. Cumulative incidence functions for each competing risk are presented in Supplementary Figure S8.

### Sensitivity analyses

A sensitivity analysis using the complete-case subset (n = 2463) was performed because the LASSO model requires complete data for variable selection. In this subset, the Full XGBoost model achieved an AUC of 0.72 (95% CI, 0.69–0.75), and the Top-20 XGBoost model yielded the same AUC. Under LOSO internal–external validation, the LASSO model demonstrated an AUC of 0.70 (95% CI, 0.67–0.73). The number of variables with non-zero coefficients per iteration had a median of 32 (IQR 30–36; range 27–43), indicating relatively stable variable selection. Supplementary Tables S4–S6 provide detailed performance measures and characteristics of the complete-case subset.

## Discussion

In this prospective, multicenter cohort study of older patients with HF, we developed and validated an ML model to predict one-year all-cause mortality. The model demonstrated acceptable discrimination and outperformed established clinical risk scores. Objective measures of physical function at discharge emerged as leading predictors. Notably, a simplified 20-variable model achieved comparable performance to the full model, offering a more feasible structure for clinical use.

Our model performed comparably to, or in some respects better than, established tools such as the SHFM and MAGGIC scores.^11^^,^^25^ The AHEAD and BIOSTAT compact scores demonstrated only moderate performance, consistent with prior Japanese studies,^25^^,^^26^ which have reported attenuated performance of Western-derived scores in this population. Conventional models often underestimate risk in older,^27^ frail patients whose clinical trajectories are difficult to predict.^28^ The prominence of the BI and SPPB in our analysis is clinically coherent. Functional impairment reflects cumulative vulnerability across multiple physiological systems,^5^^,^^29^ offering prognostic information beyond organ-specific markers. Similarly, the high ranking of serum albumin, eGFR, and C-reactive protein highlight the prognostic impact of renal dysfunction and systemic inflammation, respectively.^30^^,^^31^ The inclusion of comorbidities such as cancer illustrates how the model integrates the multi-domain markers of frailty essential for accurate risk stratification in older adults.^32^ Unlike subjective ADL assessments included in some scores,^33^ performance-based assessments such as the BI and SPPB offer greater reproducibility and capture functional limitations more directly. Thus, the SHAP-derived importance ranking aligns with clinical understanding.

ML offers methodological advantages for modeling HF in older adults, which involves non-linear and interactive relationships that are difficult for linear models to approximate. Notably, the XGBoost and LASSO performed comparably in the complete-case sensitivity analysis (AUC 0.72 vs. 0.70). This finding likely reflects the nature of the complete-case subset, which excludes patients with missing data—often those with more severe functional limitations—resulting in a more homogeneous sample where the benefits of complex model diminish. In our primary analysis on the full cohort, XGBoost's ability to accommodate non-linear effects, interactions, and informative missingness,^21^ allowed it to capture clinically meaningful patterns that linear regression models may overlook, likely contributing to its modest but consistent performance advantage. We also observed that LASSO exhibited substantial variability in variable selection across LOSO iterations, suggesting limited stability in the complete-case setting (Supplementary Tables S4–S6).

The final 20-variable model uses routinely obtainable measures and can be integrated into discharge workflows. The model's role is to shape post-discharge care strategies, not to determine discharge eligibility. It enables clinicians to identify high-risk individuals who may benefit from intensified rehabilitation, closer monitoring, or multidisciplinary care, as illustrated in our proposed clinical pathway (Supplementary Figure S9). Furthermore, the individualized risk estimates can facilitate shared decision-making with patients and families. However, successful implementation requires standardized functional assessments, periodic model recalibration, and institutional support for electronic integration. These considerations are critical for health systems serving aging populations, particularly in Asian countries, where cohorts exhibit distinct characteristics that may limit the applicability of Western-derived scores.^34^^,^^35^ Incorporating objective functional measures may therefore improve prognostic accuracy in similar settings. From a public health perspective, identifying high-risk patients at discharge can guide the efficient allocation of rehabilitation and transitional care resources. Finally, to illustrate how the 20-variable model could be incorporated into routine workflows, we implemented a simple web-based calculator that accepts routinely collected discharge variables and returns individualized 1-year risk estimates together with model-based explanations (Supplementary Figure S10). This prototype tool is intended to support risk communication and discharge planning rather than to provide a ready-to-use intervention, and its effect on clinical decision-making or patient outcomes has not yet been evaluated.

This study has several limitations. First, our model was validated using a robust LOSO framework across a nationwide Japanese cohort. While this approach demonstrates the generalizability across 96 different institutions in Japan, true external validation in geographically and clinically distinct cohorts is required to determine its applicability to non-Japanese populations. This study should be viewed as providing a robust model for this specific older Japanese population and laying the groundwork for future international validation. This is particularly relevant given that all patients were prescribed physical rehabilitation, a selection criterion that may limit applicability to the broader HF population and differ from practices in other healthcare systems. Furthermore, the recruitment period (2020–2022) coincided with the COVID-19 pandemic, which may influence generalizability. Second, our dataset lacked certain variables, such as systolic blood pressure, precluding a direct performance comparison against some established models like the SHFM. Third, our model identifies predictors, not necessarily causal factors. The inclusion of guideline-directed medical therapies raises the potential for treatment paradox, and a sensitivity analysis omitting these variables is a direction for future work. Poor physical function may be a proxy for frailty, and this study does not prove that improving function will reduce mortality. Whether targeted interventions for patients identified as high-risk by our model can improve outcomes requires testing in randomized controlled trials. Finally, while our model demonstrates high predictive accuracy, its real-world clinical utility—whether its implementation improves patient outcomes—has not yet been prospectively evaluated.

### Conclusion

In this large, nationwide cohort of older patients with HF, a ML model incorporating detailed functional measures demonstrated superior accuracy for predicting one-year mortality compared to conventional clinical risk scores. Our findings reveal that physical function at discharge is a critically important determinant of survival, rivaling the importance of traditional cardiovascular risk factors. This study underscores the essential value of integrating comprehensive geriatric and functional assessments into the routine management and risk stratification of older patients with HF.

## Contributors

**Kanji Yamada:** Conceptualization, Methodology, Software, Formal Analysis, Validation, Visualization, Writing—Original Draft, Writing—Review & Editing.

**Nobuyuki Kagiyama:** Conceptualization, Methodology, Supervision, Writing—Review & Editing.

**Tomoyuki Morisawa:** Investigation, Data Curation, Project Administration, Resources, Writing—Review & Editing.

**Masakazu Saitoh:** Investigation, Data Curation, Project Administration, Resources, Writing—Review & Editing.

**Kentaro Iwata:** Investigation, Resources, Writing—Review & Editing.

**Michitaka Kato:** Investigation, Resources, Writing—Review & Editing.

**Koji Sakurada:** Investigation, Resources, Writing—Review & Editing.

**Yuji Kono:** Investigation, Resources, Writing—Review & Editing.

**Yuki Iida:** Investigation, Resources, Writing—Review & Editing.

**Masanobu Taya:** Investigation, Resources, Writing—Review & Editing.

**Yoshinari Funami:** Investigation, Resources, Writing—Editing.

**Kentaro Kamiya:** Investigation, Supervision, Resources, Writing—Review & Editing.

**Tetsuya Takahashi:** Conceptualization, Funding Acquisition, Project Administration, Investigation, Resources, Supervision, Writing—Review & Editing.

All authors accept final responsibility for the decision to submit for publication, and take responsibility for the content of the manuscript. Kanji Yamada and Tetsuya Takahashi had full access to all the data in the study. Kanji Yamada takes responsibility for the accuracy of the data analysis. Tomoyuki Morisawa and Masakazu Saitoh verified the underlying data.

## Data sharing statement

De-identified individual participant data that underlie the results reported in this article are available to researchers affiliated with institutions participating in the J-Proof HF Registry, owing to the terms of the collaborative research agreement. Qualified investigators from participating institutions may submit a methodologically sound proposal to the corresponding author (Tetsuya Takahashi at [te-takahashi@juntendo.ac.jp]), which will be reviewed by the J-Proof HF Registry steering committee. The custom code used for model development and validation, as well as for the interactive web application, is available from the corresponding author upon reasonable written request.

## Declaration of interests

The authors declare that they have no known competing financial interests or personal relationships that could have appeared to influence the work reported in this paper.
