# Supplementary material for: Machine learning prediction of 1-year mortality in older patients with heart failure: a nationwide, multicenter, prospective cohort study
Source: Lancet Reg Health West Pac. 2026 Feb 3;67:101808. doi: 10.1016/j.lanwpc.2026.101808 (PMC12887764; doi:10.1016/j.lanwpc.2026.101808)
Supplement: Supplementary Tables and Figures [file mmc1.pdf]

Supplementary Materials – Table of Contents

I. Supplementary Methods

1. Registry Overview and Data Collection Details ···P.4
   1.1 Diagnostic Criteria and Enrollment Procedures
   1.2 Data Collection Framework
   1.3 Follow-up Procedures and Mortality Ascertainment
   1.4 Data Management and Quality Assurance
2. Outcome Adjudication Procedures ···P.5
   2.1 Definition of the Primary Outcome
   2.2 Multistep Ascertainment Process
   2.3 Classification of Cause of Death
   2.4 Timing Verification and Outlier Resolution
   2.5 Completeness of Follow-up
3. Missing Data Overview ···P.5
   3.1 Patterns of Missingness
   3.2 Use of XGBoost for Missing-Value Handling
   3.3 Complete-Case Sensitivity Analysis
4. XGBoost Model Development Details ···P.5
   4.1 Rationale for Algorithm Selection
   4.2 Model Structure and Objective Function
   4.3 Hyperparameter Optimization
   4.4 Training Workflow within the LOSO Framework
   4.5 Aggregation of Performance Across Sites
   4.6 SHAP-Based Model Interpretation
5. Performance Metric Definitions ···P.7
   5.1 Discrimination (AUC, AUPRC)
   5.2 Classification Metrics (Accuracy, Sensitivity, Specificity, PPV, NPV)
   5.3 Calibration Metrics (Calibration Plots, Brier Score)
   5.4 Reclassification Metrics (Net Reclassification Improvement)
   5.5 Decision Curve Analysis (DCA)
   5.6 Survival and Competing Risk Analyses
6. Risk Stratification Methodology ···P.8
   6.1 Derivation of Risk Groups
   6.2 Survival Analysis
   6.3 Cause-Specific Mortality and Competing Risks
   6.4 Threshold-Based Classification for NRI Analyses
   6.5 Sensitivity Analyses
7. Model Interpretability and Correlation Analysis ···P.9

7.1 SHAP-Based Interpretability
7.2 Correlation Structure Analysis
7.3 Relation to Predictor Selection

II. Supplementary Tables

1. Supplementary Table S1. …P.12
Data dictionary of all candidate predictor variables
2. Supplementary Table S2. …P.17
Reclassification and discrimination improvement for the Top-20 XGBoost model compared with established risk scores
3. Supplementary Table S3. …P.18
One-year mortality rates by predicted risk group and cause of death
4. Supplementary Table S4. …P.19
Baseline characteristics of the complete-case subset
5. Supplementary Table S5. …P.21
Performance summary in the complete-case subset
6. Supplementary Table S6. …P.22
Predictors of one-year mortality selected by LASSO regression and their corresponding odds ratios

III. Supplementary Figures

1 Supplementary Figure S1. …P.24
Flow of participants through the study
2 Supplementary Figure S2. …P.25
Calibration of the Top-20 XGBoost model for different time horizons
3 Supplementary Figure S3. …P.26
DCA based on LOSO internal–external validation
4 Supplementary Figure S4. …P.27
Calibration of the final Top-20 XGBoost model
5 Supplementary Figure S5. …P.28
DCA for the final XGBoost model
6 Supplementary Figure S6. …P.29
Correlation matrix of the top predictor variables
7 Supplementary Figure S7. …P.30
Selection of the parsimonious model
8 Supplementary Figure S8. …P.31
Cumulative incidence of death by predicted risk group and cause of death
9 Supplementary Figure S9. …P.32

Proposed clinical pathway for implementing the machine learning model in practice
10 Supplementary Figure S10. …P.33
Illustrative Workflow and User Interface of the Web-Based Prediction Tool

Supplementary Methods

1. Registry Overview and Data Collection Details

The Japanese PT Multi-center Registry of Older Frail Patients With Heart Failure (J-Proof HF) Registry is a prospective multicenter cohort designed to characterize the clinical and functional status of older adults hospitalized for heart failure (HF) in Japan. Ninety-six institutions participated Enrollment occurred from December 2020 to March 2022.

1.1 Diagnostic Criteria and Enrollment Procedures

HF was diagnosed according to Japanese Circulation Society guidelines. Consecutive patients aged ≥65 years who were hospitalized with HF and received a prescription for physical rehabilitation were screened. Standardized definitions and a unified data collection protocol were used across sites. Research staff received instruction before registry initiation.

1.2 Data Collection Framework

At admission, demographic characteristics, comorbidities, New York Heart Association (NYHA) class, laboratory tests, echocardiographic measurements, and pre-hospital functional indices (Barthel Index [BI], Kihon Checklist [KCL]) were extracted from medical records by instructed research personnel at each institution.

At discharge, licensed physical therapists performed standardized assessments, including gait speed, handgrip strength, the Short Physical Performance Battery (SPPB), limb circumferences, and ADL scales (BI and, when available, Functional Independence Measure [FIM]). All measurements and data entry were conducted by staff who received registry-specific training to ensure consistency across institutions.

1.3 Follow-up Procedures and Mortality Ascertainment

Vital status at one year was determined using a hierarchical protocol: 1) postal questionnaire, 2) telephone follow-up if no response was obtained, and 3) confirmation using medical records for patients who returned to the index institution. For confirmed deaths, the date and cause of death were obtained when available and categorized as cardiovascular (CV), non-CV, or unknown. Patients without confirmed vital status after standardized follow-up procedures were excluded.

1.4 Data Management and Quality Assurance

Data were entered locally by instructed site investigators and transmitted to the central data center. There, omissions, input errors, and out-of-range values were reviewed by an independent data manager using a secure, offline workstation with restricted access. Queries were returned to each institution for correction when necessary. After correction, all entries

were re-checked and independently verified by two licensed physical therapists to ensure accuracy and internal consistency before being finalized for analysis.

2. Outcome Adjudication Procedures

The primary outcome was all-cause mortality within one year after hospital discharge. This endpoint is widely used in HF prognostic studies and reflects the multimorbidity profile of older adults. Cause of death was categorized as following; 1) CV deaths, 2) non-CV deaths, and 3) unknown. CV deaths included worsening HF. Unknown cause was assigned when documentation was insufficient. Vital status and dates of death were validated using available sources whenever possible.

3. Missing Data Overview

3.1 Patterns of Missingness

Missingness arose from inter-institutional differences in testing and from patient-related limitations. Performance-based measures such as gait speed and SPPB were frequently missing in patients unable to complete testing, reflecting severe frailty. Laboratory and cognitive measures were missing at institutions where they were not routinely performed. The extent of missingness for each variable is summarized in Supplementary Table S1.

3.2 Use of XGBoost for Missing-Value Handling

We did not apply imputation because missingness in this population is unlikely to be random and may carry prognostic information. eXtreme Gradient Boosting (XGBoost) handles missing data natively by learning, at each split, the direction ("default branch") that optimizes the objective function when a value is missing. This allows the model to incorporate informative missingness during both training and prediction.

3.3 Complete-Case Sensitivity Analysis

To examine the impact of missingness, a complete-case subset (n = 2,463) was used to develop and evaluate a Least Absolute Shrinkage and Selection Operator (LASSO) logistic regression model under the same leave-one-site-out (LOSO) framework. Both XGBoost and LASSO models showed reduced discrimination in the complete-case analysis, suggesting that missingness contained clinically relevant information. This supported the use of XGBoost on the full dataset without imputation.

4. XGBoost Model Development Details

4.1 Rationale for Algorithm Selection

XGBoost was chosen because it models non-linear relationships and interactions, accommodates high-dimensional tabular data, and handles missing values without explicit

imputation. These properties are advantageous in older patients with multimorbidity and heterogeneous functional profiles.

4.2 Model Structure and Objective Function

Models were trained using gradient-boosted decision trees with logistic loss and L2 regularization. Early stopping and a fixed learning rate were used to prevent overfitting. This structure allowed flexible modeling of complex patterns while maintaining stable generalization performance.

4.3 Hyperparameter Optimization

Hyperparameters were tuned within each LOSO training set using 5-fold cross-validation. The search included:

- ✓ max_depth
- ✓ min_child_weight
- ✓ eta (learning rate)
- ✓ subsample
- ✓ colsample_bytree
- ✓ gamma
- ✓ lambda (L2 regularization)

A grid search with early stopping was used, and the parameter set minimizing cross-validated log-loss was selected. These values were then fixed when retraining the model on the full training portion of each LOSO iteration to maintain consistency across folds.

4.4 Training Workflow within the LOSO Framework

For each LOSO iteration:

1. One site was held out as an independet test set.
2. Hyperparameters were tuned on the remaining sites.
3. The final model was retrained on all training sites using the optimized parameters.
4. Early stopping was applied if validation performance did not improve within 50 rounds.
5. Predictions were generated only for the held-out site.

This iterative process provided 96 fully out-of-sample evaluations, ensuring that performance reflected generalizability across institutions with different patient profiles.

4.5 Aggregation of Performance Across Sites

Performance metrics—Area Under the Receiver Operating Characteristic Curve (AUC), Area Under the Precision–Recall Curve (AUPRC), calibration, and decision-curve net benefit—were calculated for each held-out site and aggregated using macro-means. Bootstrapped 95% confidence intervals were generated across site-level results. This approach avoided optimistic bias and reflected real-world variability across institutions.

4.6 SHAP-Based Model Interpretation

SHapley Additive exPlanations (SHAP) values were computed only in held-out LOSO test sets to ensure interpretability reflected out-of-sample behavior. For each fold, 100 Monte Carlo permutations were used to approximate SHAP values. Aggregated mean absolute SHAP values were used to derive global feature importance rankings, which informed selection of the parsimonious Top-20 model. Individual-level SHAP plots were used to visualize patient-specific predictors for model deployment.

5. Performance Metric Definitions

This section summarizes the metrics used to evaluate discrimination, calibration, classification performance, and clinical utility. All metrics were calculated for each LOSO test set and aggregated across sites.

5.1 Discrimination

Area Under the ROC Curve (AUC)

AUC quantifies the probability that a randomly selected patient who died within 1 year receives a higher predicted risk than a survivor.

Area Under the Precision–Recall Curve (AUPRC)

AUPRC is informative in imbalanced datasets, summarizing the relationship between sensitivity and positive predictive value across thresholds.

5.2 Classification Metrics

Computed at the threshold that maximized Youden's Index, unless otherwise stated.

- ✓ Accuracy: proportion of correctly classified patients.
- ✓ Sensitivity: proportion of deaths correctly identified.
- ✓ Specificity: proportion of survivors correctly identified.
- ✓ PPV (Precision): probability of death among those predicted to die.
- ✓ NPV: probability of survival among those predicted to survive.

5.3 Calibration Metrics

Calibration Plots

Predicted risks were grouped into deciles, and observed event rates were compared graphically with model-predicted probabilities.

Brier Score

Represents the mean squared difference between predicted risk and observed outcome. Lower scores indicate better combined calibration and discrimination.

5.4 Reclassification Metrics

Net Reclassification Improvement (NRI)

NRI quantified the improvement in classification offered by the Top-20 XGBoost model relative to benchmark scores (AHEAD, BIOSTAT compact).

A single clinically relevant threshold (20% predicted risk) was used:

- ✓ "Upward reclassification" means moving from below to above the threshold.
- ✓ NRI combines improvements for events (deaths) and nonevents (survivors).

Event and nonevent contributions were reported separately.

5.5 Decision Curve Analysis (DCA)

DCA assessed the clinical utility of using model-based predictions across a range of threshold probabilities.

A model was considered clinically useful if its net benefit exceeded that of the default strategies of treating:

- ✓ all patients
- ✓ no patients

5.6 Survival and Competing Risk Analyses

Survival analyses were conducted to assess the prognostic validity of model-based risk groups:

- ✓ Kaplan–Meier curves compared overall survival across tertiles of predicted risk.
- ✓ Log-rank tests assessed statistical significance.
- ✓ Competing risks analyses estimated cause-specific cumulative incidence for CV and non-CV deaths using the alternative cause as a competing event.

6. Risk Stratification Methodology

Risk stratification was performed using predicted 1-year mortality from the final 20-predictor XGBoost model. The aim was to examine whether model-based risk estimates aligned with clinically meaningful gradients in mortality and causes of death.

6.1 Derivation of Risk Groups

Patients were divided into three groups based on tertiles of predicted 1-year mortality:

- ✓ Low risk: lowest tertile
- ✓ Intermediate risk: middle tertile
- ✓ High risk: highest tertile

Tertiles were selected to provide balanced group sizes and to avoid outcome-dependent cut-points. No survival information was used to define these groups.

6.2 Survival Analysis

Kaplan–Meier curves were constructed using time since discharge as the time scale.
The log-rank test compared survival across the three groups.
Patients were censored at the date last known alive if they did not experience the outcome within 1 year.

6.3 Cause-Specific Mortality and Competing Risks

To evaluate the relationship between predicted risk and mode of death:

1. CV vs. non-CV death distributions were calculated within each tertile among patients who died.
2. Competing risks analyses quantified cumulative incidence:
   CV death treating non-CV death as a competing event
   Non-CV death treating CV death as a competing event

This approach prevented overestimation of cumulative incidence by accounting for mutually exclusive events.

6.4 Threshold-Based Classification for NRI Analyses

For reclassification analyses, a predefined threshold of 20% predicted 1-year mortality was applied:

- ✓ High-risk: ≥20%
- ✓ Low-Risk: <20%

This threshold was applied consistently across models to compute NRI.

6.5 Sensitivity Analyses

Robustness was evaluated using:

- ✓ decile-based risk groups, and
- ✓ alternative thresholds (e.g., Youden's Index) for classification metrics.

These analyses yielded consistent results and did not materially alter the observed patterns in mortality or risk discrimination.

7. Model Interpretability and Correlation Analysis

Model interpretability was examined using SHAP and correlation structure analysis. These complementary approaches assessed how individual predictors contributed to model output and whether relationships among variables were consistent with clinical expectations.

7.1 SHAP-Based Interpretability

Computation of SHAP Values

SHAP values were computed using the fastshap package. To avoid information leakage, values were calculated only for patients in the held-out site for each LOSO iteration. For each iteration:

1. The final model was applied to all patients in the held-out site.
2. SHAP values were estimated using 100 Monte Carlo permutations.
3. Values were standardized for aggregation across folds.

This procedure ensured that interpretability reflected true out-of-sample performance.

Global Importance Ranking

Mean absolute SHAP values across LOSO folds provided a global importance ranking. This ranking was used to:

- ✓ identify predictors consistently contributing to model performance, and
- ✓ guide selection of variables for the 20-predictor model in conjunction with clinical review.

The ranking captured non-linear and interaction effects not detectable with linear methods.

Individual-Level Explanations

Individual SHAP plots illustrated how each predictor shifted a patient's risk estimate relative to the baseline risk. These plots support model transparency for potential clinical implementation.

7.2 Correlation Structure Analysis

Spearman correlation coefficients were calculated for the top 30 predictors (based on SHAP ranking). Pairwise-complete observations were used to allow inclusion of variables with partial missingness. Hierarchical clustering (complete linkage) arranged predictors according to similarity in correlation patterns, displayed as a lower-triangle correlation matrix (Supplementary Figure S6).

Interpretation

The analysis served to:

1. Identify clusters of clinically related predictors
   - ✓ functional measures: SPPB, gait speed, grip strength
   - ✓ renal function markers: creatinine, blood urea nitrogen, estimated glomerular filtration rate (eGFR)
   - ✓ HF severity indicators: natriuretic peptides, E/e', left atrial dimensions
2. Confirm appropriate distribution of importance in XGBoost, which can incorporate correlated variables without the instability seen in linear models.

7.3 Relation to Predictor Selection

Correlation patterns and SHAP-derived importance were jointly reviewed by a multidisciplinary team. Predictors were retained in the final model when they:

- ✓ showed consistent contribution across LOSO folds,
- ✓ represented clinically distinct constructs, and
- ✓ avoided redundancy when strong correlations were identified.

This combined approach supported an interpretable and clinically coherent model without compromising discriminative performance.

Supplementary Table S1. Data dictionary of all candidate predictor variables

Definition and Measurement	Type	Unit	Handling	Missing Values, n (%)
Basic Info				
Age at hospital admission	Continuous	years	Used as is	0 (0.0)
Biological sex	Binary	1=Male, 0=Female	Used as is	0 (0.0)
BMI at admission,	Continuous	kg/m^2	Derived	318 (3.3)
BMI at discharge	Continuous	kg/m^2	Derived	337 (3.5)
Current smoking status at admission	Binary	1=Yes, 0=No	Used as is	10 (0.1)
BI prior to admission	Continuous	0-100 points	Used as is	201 (2.1)
KCL total score prior to admission	Continuous	0-25 points	Used as is	1,106 (11.4)
Patient's living arrangement and social support status prior to admission.	Categorical	0=Alone without support, 1=Alone with support, 2=Not alone without support, 3=Not alone with support	Used as is	20 (0.2)
HF-Related				
Etiology of HF: Ischemic Heart Disease	Binary	1=Yes, 0=No	Used as is	1 (0.0)
Etiology of HF: Cardiomyopathy	Binary	1=Yes, 0=No	Used as is	1 (0.0)
Etiology of HF: Arrhythmia	Binary	1=Yes, 0=No	Used as is	1 (0.0)
Etiology of HF: Valvular Heart Disease	Binary	1=Yes, 0=No	Used as is	1 (0.0)
History of prior hospitalization for HF	Binary	1=Yes, 0=No	Used as is	4 (0.0)
NYHA at admission	Categorical	I, II, III, IV	Used as is	24 (0.2)
Clinical Scenario on admission	Categorical	1, 2, 3, 5	Used as is	21 (0.2)
LVEF	Continuous	%	Used as is	589 (6.1)
LAD	Continuous	mm	Used as is	1,006 (10.4)
E/e’	Continuous	Ratio	Used as is	2,677 (27.6)

Definition and Measurement	Type	Unit	Handling	Missing Values, n (%)
Comorbidities				
History of Hypertension	Binary	1=Yes, 0=No	Used as is	0 (0.0)
History of Diabetes Mellitus	Binary	1=Yes, 0=No	Used as is	0 (0.0)
History of Hyperlipidemia	Binary	1=Yes, 0=No	Used as is	0 (0.0)
History of Chronic Kidney Disease	Binary	1=Yes, 0=No	Used as is	0 (0.0)
History of Hemodialysis	Binary	1=Yes, 0=No	Used as is	0 (0.0)
History of Angina Pectoris	Binary	1=Yes, 0=No	Used as is	0 (0.0)
History of Myocardial Infarction	Binary	1=Yes, 0=No	Used as is	0 (0.0)
History of COPD	Binary	1=Yes, 0=No	Used as is	0 (0.0)
History of Musculoskeletal Disease	Binary	1=Yes, 0=No	Used as is	0 (0.0)
History of Cerebrovascular Accident	Binary	1=Yes, 0=No	Used as is	0 (0.0)
History of Cancer	Binary	1=Yes, 0=No	Used as is	0 (0.0)
History of Peripheral Artery Disease	Binary	1=Yes, 0=No	Used as is	0 (0.0)
History of Atrial Fibrillation or Flutter	Binary	1=Yes, 0=No	Used as is	0 (0.0)
Blood Test at admission				
Hemoglobin level	Continuous	g/dL	Used as is	24 (0.2)
Serum albumin level	Continuous	g/dL	Used as is	256 (2.6)
Serum sodium level	Continuous	mEq/L	Used as is	26 (0.3)
eGFR	Continuous	mL/min/1.73m^2	Used as is	109 (1.1)
Blood Urea Nitrogen level	Continuous	mg/dL	Used as is	21 (0.2)
Serum creatinine level	Continuous	mg/dL	Log-transformed	9 (0.1)
C-reactive protein level	Continuous	mg/dL	Log-transformed	449 (4.6)

Definition and Measurement	Type	Unit	Handling	Missing Values, n (%)
Combined natriuretic peptide level	Continuous	Standardized Z-score	Log-transformed, standardized, and combined BNP/NT-proBNP values	189 (1.9)
Medication at discharge				
Prescription of Beta-blocker	Binary	1=Yes, 0=No	Used as is	12 (0.1)
Prescription of ACE-I/ARB/ARNI	Binary	1=Yes, 0=No	Used as is	27 (0.3)
Prescription of MRA	Binary	1=Yes, 0=No	Used as is	12 (0.1)
Prescription of Calcium Channel Blocker	Binary	1=Yes, 0=No	Used as is	12 (0.1)
Prescription of SGLT-2 Inhibitor	Binary	1=Yes, 0=No	Used as is	25 (0.3)
Prescription of Diuretic	Binary	1=Yes, 0=No	Used as is	12 (0.1)
Prescription of Antiarrhythmic drug	Binary	1=Yes, 0=No	Used as is	12 (0.1)
Prescription of Nitrate	Binary	1=Yes, 0=No	Used as is	12 (0.1)
Prescription of Anticoagulant	Binary	1=Yes, 0=No	Used as is	12 (0.1)
Prescription of Antiplatelet	Binary	1=Yes, 0=No	Used as is	13 (0.1)
Prescription of HCN Channel Blocker	Binary	1=Yes, 0=No	Used as is	25 (0.3)
Treatment				
History of cardiac device implantation (PM, ICD, CRT)	Binary	1=Yes, 0=No	Used as is	12 (0.1)
History of PCI	Binary	1=Yes, 0=No	Used as is	12 (0.1)
History of Catheter Ablation	Binary	1=Yes, 0=No	Used as is	12 (0.1)
History of Direct Current Cardioversion	Binary	1=Yes, 0=No	Used as is	12 (0.1)
Physical therapy during hospitalization				
Total units of physical therapy	Continuous	units (1 unit=20 min)	Used as is	0 (0.0)
ADL training performed	Binary	1=Yes, 0=No	Used as is	8 (0.1)
Gait training performed	Binary	1=Yes, 0=No	Used as is	9 (0.1)

Definition and Measurement	Type	Unit	Handling	Missing Values, n (%)
Resistance training performed	Binary	1=Yes, 0=No	Used as is	13 (0.1)
Endurance training performed	Binary	1=Yes, 0=No	Used as is	16 (0.2)
Machine training performed	Binary	1=Yes, 0=No	Used as is	18 (0.2)
Functional status				
Grip strength at start of rehabilitation	Continuous	kg	Used as is	1,483 (15.3)
Grip strength at discharge	Continuous	kg	Used as is	1,428 (14.7)
BI at discharge	Continuous	0-100 points	Used as is	235 (2.4)
FIM at discharge	Continuous	18-126 points	Used as is	3,858 (39.8)
Presence of cognitive impairment	Binary	1=Yes, 0=No	Derived	854 (8.8)
Maximum gait speed at discharge	Continuous	m/s	Used as is	2,729 (28.1)
Comfortable gait speed at discharge	Continuous	m/s	Used as is	2,116 (21.8)
Maximum upper arm circumference at discharge	Continuous	cm	Used as is	1,501 (15.4)
Maximum calf circumference at discharge	Continuous	cm	Used as is	1,498 (15.4)
SPPB total score at discharge	Continuous	0-12 points	Used as is	18 (0.2)
SPPB balance sub-score	Continuous	0-4 points	Derived	1,327 (13.7)
SPPB gait speed sub-score	Continuous	0-4 points	Derived	1,362 (14.0)
SPPB chair stand sub-score	Continuous	0-4 points	Derived	1,367 (14.1)
Japanese CHS criteria total score	Continuous	0-5 points	Used as is	3,256 (33.6)
Others				
Length of hospital stay	Continuous	days	Used as is	0 (0.0)
Discharged to home	Binary	1=Yes, 0=No	Used as is	0 (0.0)
Prescription for outpatient cardiac rehabilitation	Binary	1=Yes, 0=No	Used as is	144 (1.5)

Definition and Measurement	Type	Unit	Handling	Missing Values, n (%)

Abbreviations: ACEI, angiotensin-converting enzyme inhibitor; ARB, angiotensin II receptor blocker; ARNI, angiotensin receptor neprilysin inhibitor; BI, Barthel Index; BMI, Body Mass Index; CRT, Cardiac Resynchronization Therapy; CHS, Cardiovascular Health Study; COPD, Chronic obstructive pulmonary disease; E/e', ratio of early mitral inflow velocity to mitral annular early diastolic velocity; eGFR, Estimated Glomerular Filtration Rate; FIM, Functional Independence Measure; HF, Heart Failure; ICD, Implantable Cardioverter-Defibrillator; KCL, Kihon Checklist; LAD, Left Atrial Diameter; LVEF, Left Ventricular Ejection Fraction; MRA, Mineralocorticoid Receptor Antagonist; NYHA, New York Heart Association; PCI, Percutaneous Coronary Intervention; PM, Pacemaker; SPPB, Short Physical Performance Battery.

Supplementary Table S2. Reclassification and discrimination improvement for the Top-20 XGBoost model compared with established risk scores

Comparison	Metric	Estimate (95% CI)
vs. AHEAD score		
	Categorical NRI (Overall)	21.3% (17.8% to 24.7%)
	Events (cases)	16.3% (12.8% to 20.0%)
	Non-events (controls)	5.0% (2.8% to 6.7%)
vs. BIOSTAT compact		
	Categorical NRI (Overall)	24.0% (20.7% to 27.5%)
	Events (cases)	28.3% (25.0% to 32.1%)
	Non-events (controls)	-4.3% (-5.8% to -2.7%)

Footnote: Categorical NRI was calculated using a prespecified 20% risk threshold to define high- and low-risk groups. This threshold was chosen for its clinical relevance and was consistent with the optimal cut-point derived from the learning cohort using Youden’s index. It was used only for reclassification analyses and not during model training. A positive NRI indicates improved reclassification, and statistical significance is inferred when the 95% CI does not include zero.
Abbreviations: CI, Confidence Interval; NRI, Net Reclassification Improvement.

Supplementary Table S3. One-year mortality rates by predicted risk group and cause of death

Risk Group	N of Patients (% of Total)	Total Events (% Event Rate)	CV Deaths (% of Patients)	Non-CV Deaths (% of Patients)	Unknown (% of Patients)
Low Risk	3,234 (33.3)	82 (2.5)	43 (1.3)	38 (1.2)	1 (0.0)
Intermediate Risk	3,233 (33.3)	371 (11.5)	173 (5.4)	193 (6.0)	5 (0.2)
High Risk	3,233 (33.3)	1,148 (35.5)	596 (18.4)	538 (16.6)	14 (0.4)
Total	9,700 (100.0)	1,601 (16.5)	812 (8.4)	769 (7.9)	20 (0.2)

Footnote: Patients were stratified into three equally sized risk groups according to the predicted probability of 1-year all-cause mortality. Event rates were calculated as the number of deaths in each category divided by the total number of patients within that group. The distribution of event rates across the low-, intermediate-, and high-risk groups reflects the model's ability to separate patients with markedly different prognoses. Among the 812 CV deaths, 719 (88.5%) were attributed to worsening HF.

Abbreviations: CV, Cardiovascular; HF, heart failure.

Supplementary Table S4. Baseline characteristics of the complete-case subset

Characteristic	Overall
Number of facilities, n	61
Number of patients, n	2,463
Demographic data	
Age, years	82 [76, 87]
Male, n (%)	1,299 (52.7)
BMI, kg/m^2	21.1 [18.7, 23.6]
Prehospital BI, points	100 [90, 100]
Prehospital KCL, points	11 [7, 14]
Medical history	
Etiology of HF, n (%)	
Ischemic heart disease	761 (30.9)
Cardiomyopathic	313 (12.7)
Arrhythmia	1,142 (46.3)
Valvular	892 (36.2)
NYHA, n (%)	
I/II	588 (23.9)
III/IV	1,875 (76.1)
History of HF hospitalization, n (%)	918 (37.3)
Comorbidities, n (%)	
Hypertension	1,723 (70.0)
Diabetes Mellitus	909 (36.9)
Hyperlipidemia,	820 (33.3)
Chronic Kidney Disease	1,015 (41.2)
COPD	200 (8.1)
Cancer	399 (16.2)
Echocardiography	
LVEF, %	48 [34, 60]
LAD, mm	44 [39, 48]
E/e’	17.2 [12.7, 23.6]
Blood tests	
Albumin, g/dL	3.6 [3.2, 3.8]
eGFR, mL/min/1.73m2	43 [28, 56]
C-reactive protein, mg/dL	0.40 [0.14, 1.44]
Hemoglobin, g/dL	12 [10, 13]
Na, mEq/L	140 [138, 142]
BNP, pg/mL	549 [311, 980]

NT-proBNP, pg/mL	4,748 [2,106, 9,799]
Medications, n (%)	
ACE-I/ARB/ARNI	1,437 (58.3)
β-blockers	1,711 (69.5)
MRA	798 (32.4)
SGLT-2 inhibitor	396 (16.1)
Functional characteristics	
Cognitive decline, n (%)	842 (34.2)
BI at discharge, points	95 [85, 100]
Grip strength at discharge, kg	19 [14, 25]
Maximum walking speed, m/s	0.99 [0.73, 1.24]
Maximum leg circumference, cm	31 [28, 33]
SPPB, points	9 [6, 11]

Footnote: Values are presented as median [interquartile range] or as percentages for the complete-case subset (n = 2,463). This subset includes patients with no missing data across all candidate predictors and therefore represents a healthier and more functionally complete group compared with the overall cohort.

Abbreviations: ACEI, angiotensin-converting enzyme inhibitor; ARB, angiotensin II receptor blocker; ARNI, angiotensin receptor neprilysin inhibitor; BI, Barthel Index; BMI, body mass index; BNP, brain natriuretic peptide; COPD, chronic obstructive pulmonary disease; eGFR, estimated glomerular filtration rate; E/e’, ratio of early mitral inflow velocity to mitral annular early diastolic velocity; HF, heart failure; KCL, Kihon Check List; LAD, left atrial diameter; LVEF, left ventricular ejection fraction; MRA, mineralocorticoid receptor antagonist; Na, sodium; NT-pro BNP, N-terminal pro brain natriuretic peptide; NYHA, New York Heart Association functional classification; SGLT-2, sodium-glucose co-transporter 2; SPPB, Short physical performance battery.

Supplementary Table S5. Performance summary in the complete-case subset

Model	AUC (95% CI)	AUPRC	Accuracy	Sensitivity	Specificity	PPV	NPV
XGBoost (Full)	0.72 (0.69 to 0.75)	0.25	0.70	0.66	0.70	0.24	0.93
XGBoost (Top-20)	0.72 (0.69 to 0.75)	0.26	0.61	0.76	0.59	0.21	0.94
LASSO	0.70 (0.67 to 0.73)	0.22	0.68	0.64	0.68	0.23	0.93
AHEAD score	0.58 (0.55 to 0.62)	0.17	0.47	0.68	0.44	0.15	0.91
BIOSTAT compact	0.62 (0.59 to 0.65)	0.18	0.56	0.64	0.55	0.17	0.91

Foot note: Performance was evaluated in the complete-case subset (n = 2,463), which included only patients without missing data across all candidate predictors. This subset enables a direct comparison with the LASSO model, which requires fully observed data and may therefore represent a healthier and more completely assessed group than the overall cohort. Accuracy, sensitivity, specificity, PPV, and NPV were calculated at the optimal probability threshold determined by maximizing Youden's index. In the LOSO internal–external validation of the LASSO model, the number of predictors with non-zero coefficients per iteration had a median of 32 (IQR 30–36; range 27–43), indicating substantial variability in variable selection.

Abbreviations: AUC, Area Under the Receiver Operating Characteristic Curve; CI, Confidence Interval; LASSO, Least Absolute Shrinkage and Selection Operator; LOSO, leave-one-site-out;NPV, Negative Predictive Value; PPV, Positive Predictive Value; XGBoost, eXtreme Gradient Boosting.

Supplementary Table S6. Predictors of one-year mortality selected by LASSO regression and their corresponding odds ratio

Feature	OR	95% CI
Age	1.02	1.00-1.04
Sex (Male)	1.47	1.15-2.29
Comorbidity: Ischemic Heart Disease	1.02	1.00-1.51
Comorbidity: Cardiomyopathy	1.04	1.00-1.66
History of HF Hospitalization	1.16	1.00-1.47
Clinical Scenario at Admission	1.17	1.00-1.40
Comorbidity: Hypertension	0.92	0.68-1.00
Comorbidity: Chronic Kidney Disease	1.18	1.00-1.60
Comorbidity: Angina Pectoris	1.04	0.92-1.48
Comorbidity: Musculoskeletal Disease	1.07	1.00-1.50
Comorbidity: Cancer	1.62	1.23-2.30
Comorbidity: Atrial Fibrillation or Flutter	1.07	1.00-1.50
Left Atrial Diameter	1.00	1.00-1.02
E/e'	1.01	1.00-1.02
Albumin	0.85	0.62-1.00
Blood Urea Nitrogen	1.01	1.00-1.02
Sodium	0.98	0.95-1.00
Creatinine	1.07	1.00-2.03
Natriuretic peptide	1.02	1.00-1.20
Patient's living arrangement and social support status	1.01	0.99-1.17
Kihon Checklist (Pre-hospital)	1.02	1.00-1.05
Grip Strength at Start of Rehabilitation	0.99	0.97-1.00
Machine training performed	0.91	0.36-1.00
Prescription of Calcium Channel Blocker	0.88	0.61-1.00
Prescription of Nitrate	1.12	1.00-1.80
History of cardiac device implantation (PM, ICD, CRT)	0.66	0.31-0.99
History of Direct Current Cardioversion	0.91	0.21-1.00
BMI at Discharge	0.97	0.91-1.00
BI at discharge	1.00	0.99-1.00
FIM at Discharge	0.99	0.98-1.00
Japanese CHS criteria total score	1.06	1.00-1.21
Maximum Upper Arm Circumference	0.96	0.90-1.00
Maximum leg circumference	0.97	0.92-1.00
SPPB (Gait Subscore)	0.87	0.72-1.00
SPPB (Chair Stand Subscore)	0.97	0.88-1.00

Footnote: The table lists all predictors with non-zero coefficients selected by the LASSO regression model, developed using the complete-case subset (n = 2,463). To facilitate clinical interpretation, OR and 95% confidence intervals were estimated by refitting a standard multivariable logistic regression model including only the variables selected by LASSO. These OR estimates describe associations conditional on the selected feature set and do not represent the penalized coefficients from the original LASSO model. This subset includes only patients with complete information for all candidate predictors and may therefore differ from the overall cohort in clinical characteristics.

Abbreviations: BI, Barthel Index; BMI, body mass index; CHS, Cardiovascular Health Study; CI, confidence intervals; COPD, chronic obstructive pulmonary disease; eGFR, estimated glomerular filtration rate; E/e', ratio of early mitral inflow velocity to mitral annular early diastolic velocity; FIM, Functional Independence Measure; LASSO, Least Absolute Shrinkage and Selection Operator; OR, Odds ratio; SPPB, Short physical performance battery.

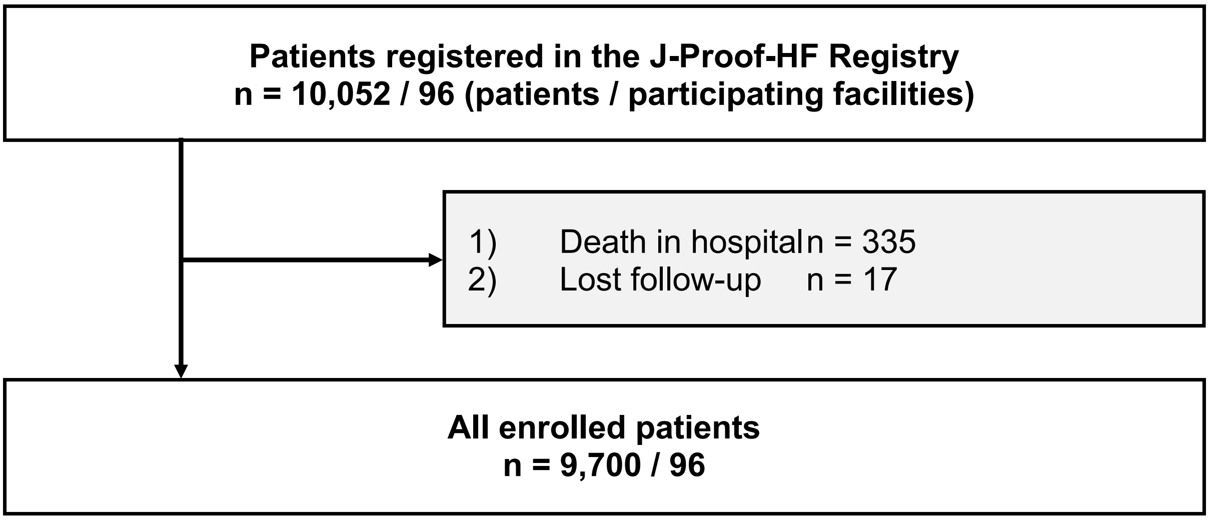

Supplementary Figure S1. Flow of participants through the study

Footnote: The diagram shows patient screening and enrollment from the J-Proof HF Registry and the reasons for exclusion that led to the final analytical cohort. In the figure, “n” denotes the number of patients, and the values after the slash indicate the number of participating facilities.

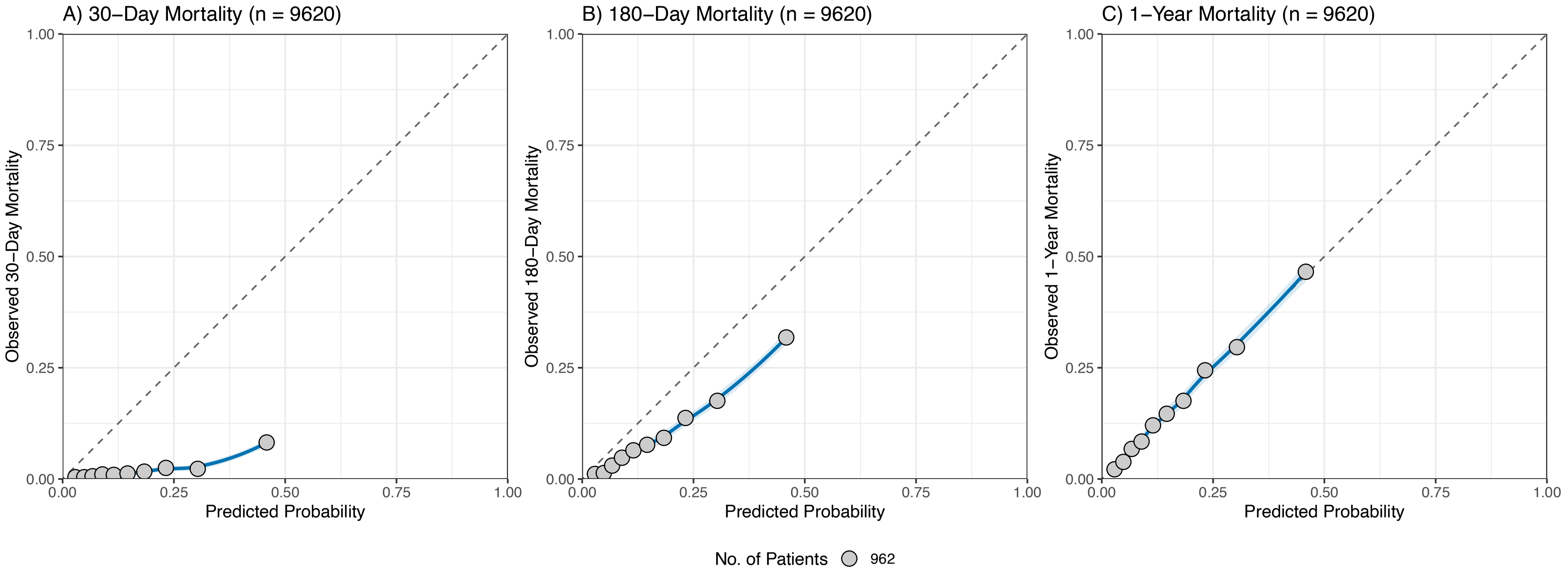

Supplementary Figure S2. Calibration of the Top-20 XGBoost model for different time horizons

Footnote: The figure presents calibration plots evaluating how the 1-year Top-20 XGBoost model performs when its predicted probabilities are compared with observed mortality at different time horizons. Panels show calibration for (A) 30-day mortality, (B) 180-day mortality, and (C) the primary endpoint, 1-year mortality. The predicted probabilities on the x-axis are generated from the original 1-year model for all plots. The dashed diagonal line denotes perfect calibration. The model demonstrates good calibration for 1-year mortality but shows systematic overestimation when applied to shorter-term outcomes, particularly 30-day mortality. For LOSO internal–external validation, test sites with fewer than 10 patients or with single-class outcomes were excluded, resulting in 9,620 patients included in the calibration analyses.

Abbreviations: LOSO, leave-one-site-out; XGBoost, eXtreme Gradient Boosting.

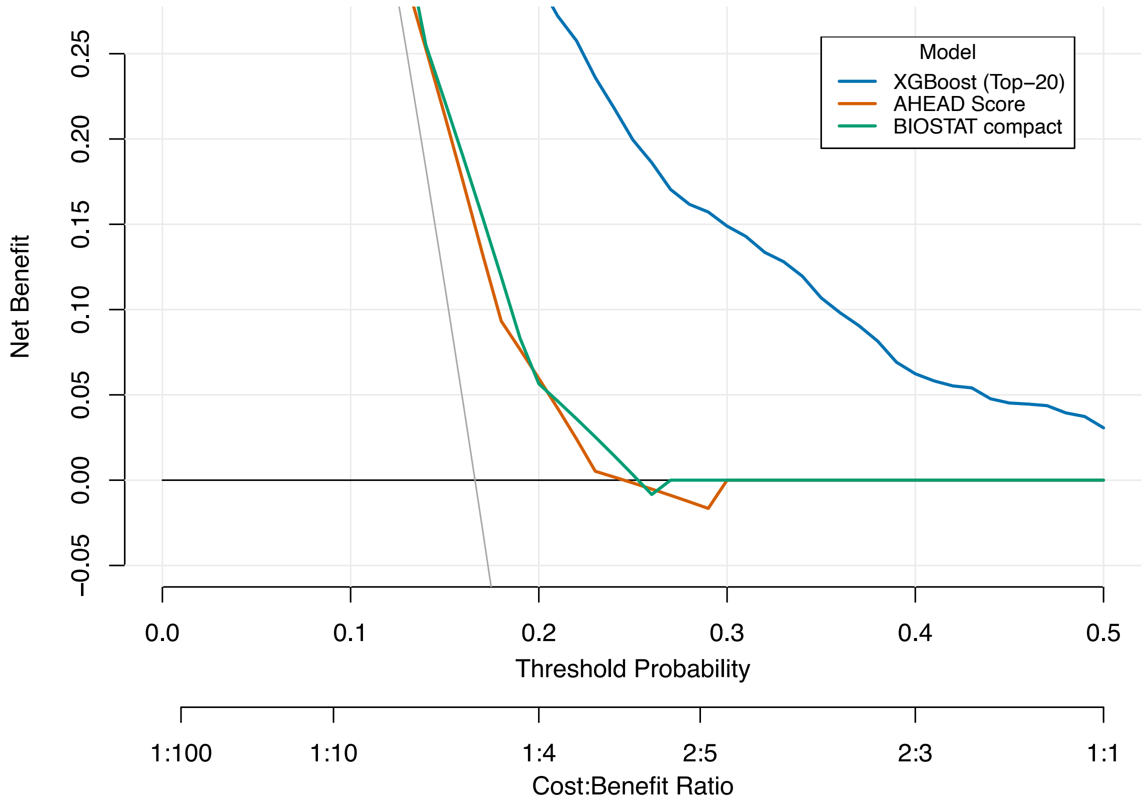

Supplementary Figure S3. DCA based on LOSO internal–external validation

Footnote: The figure shows the decision curve analysis for the Top-20 XGBoost model during leave-one-site-out internal–external validation, compared with the AHEAD and BIOSTAT compact scores. Net benefit is plotted across a range of threshold probabilities to evaluate model utility when applied to unseen sites. The XGBoost model demonstrates higher net benefit than the benchmark scores within clinically relevant thresholds (approximately 10–40%). Grey curves represent the treat-all and treat-none strategies. Sites with fewer than 10 patients or with single-class outcomes were excluded, yielding 9,620 patients for the LOSO analysis.

Abbreviations: DCA, Decision Curve Analysis; LOSO, leave-one-site-out; XGBoost, eXtreme Gradient Boosting.

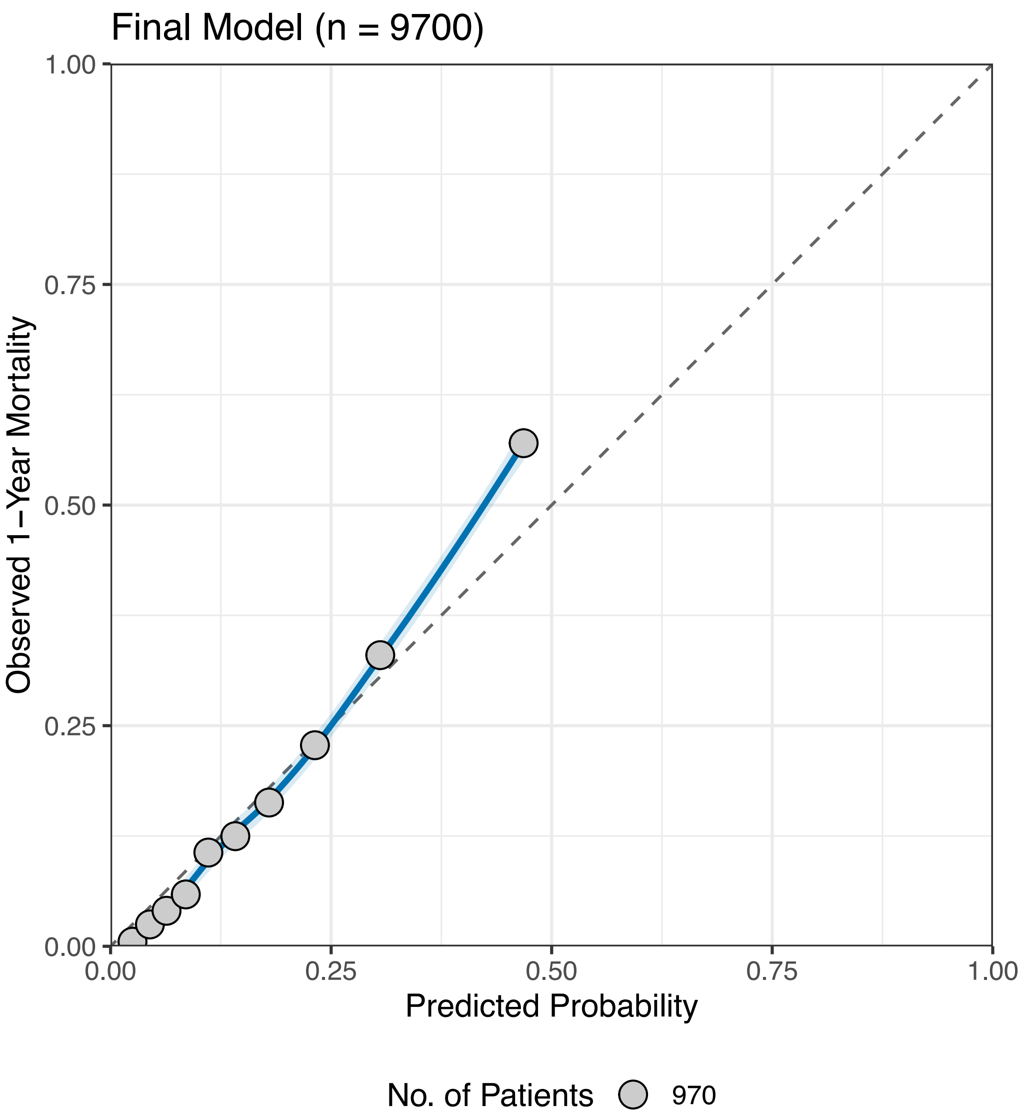

Supplementary Figure S4. Calibration of the final Top-20 XGBoost model

Footnote: The figure presents the calibration performance of the final Top-20 XGBoost model for predicting 1-year all-cause mortality in the full cohort (n = 9,700). Predicted probabilities are shown on the x-axis and observed event rates within deciles of predicted risk on the y-axis. The dashed diagonal line indicates perfect calibration. The plot demonstrates that the model shows good agreement between predicted and observed risks across the range of predicted probabilities.

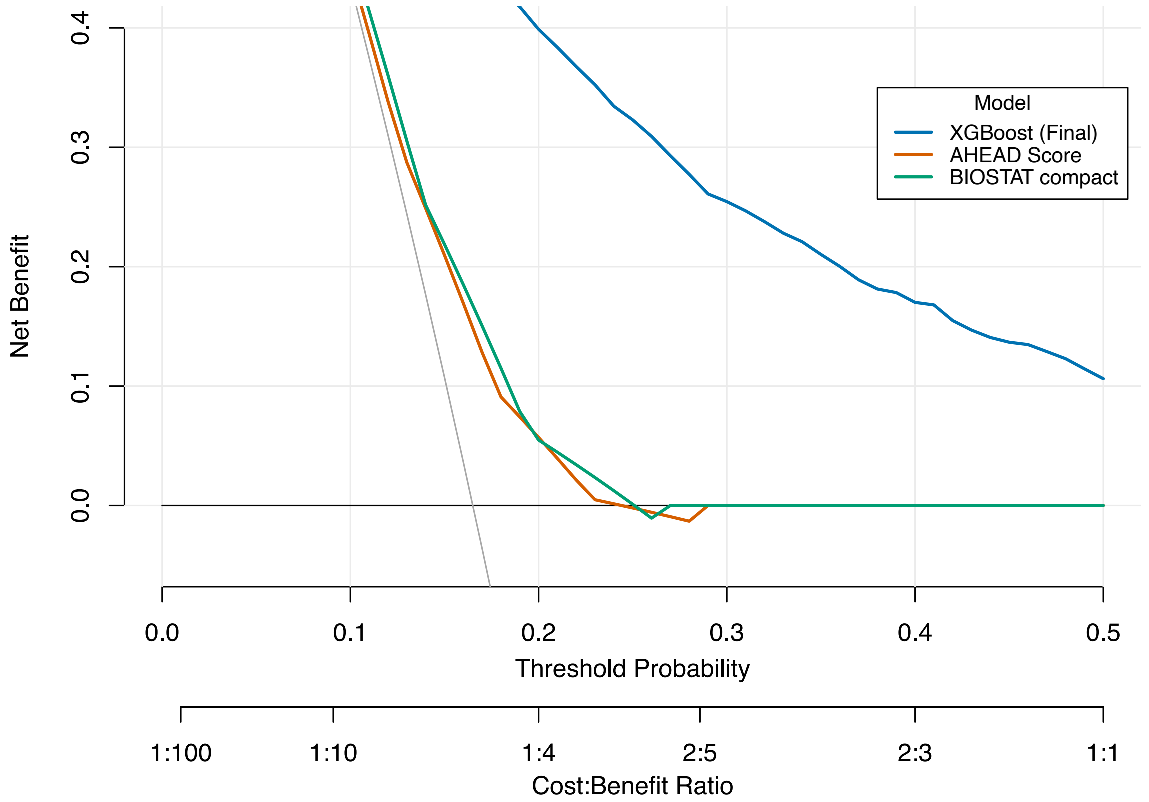

Supplementary Figure S5. DCA for the final XGBoost model

The figure presents the decision curve analysis for the final XGBoost model, retrained on the entire cohort, compared with the AHEAD and BIOSTAT compact scores. The plot illustrates net benefit across threshold probabilities for the final XGBoost model intended for clinical application. The final XGBoost model maintains greater net benefit than the benchmark scores within clinically relevant thresholds (approximately 10–40%). Grey curves indicate the treat-all and treat-none strategies.

Abbreviations: DCA, Decision Curve Analysis; XGBoost, eXtreme Gradient Boosting.

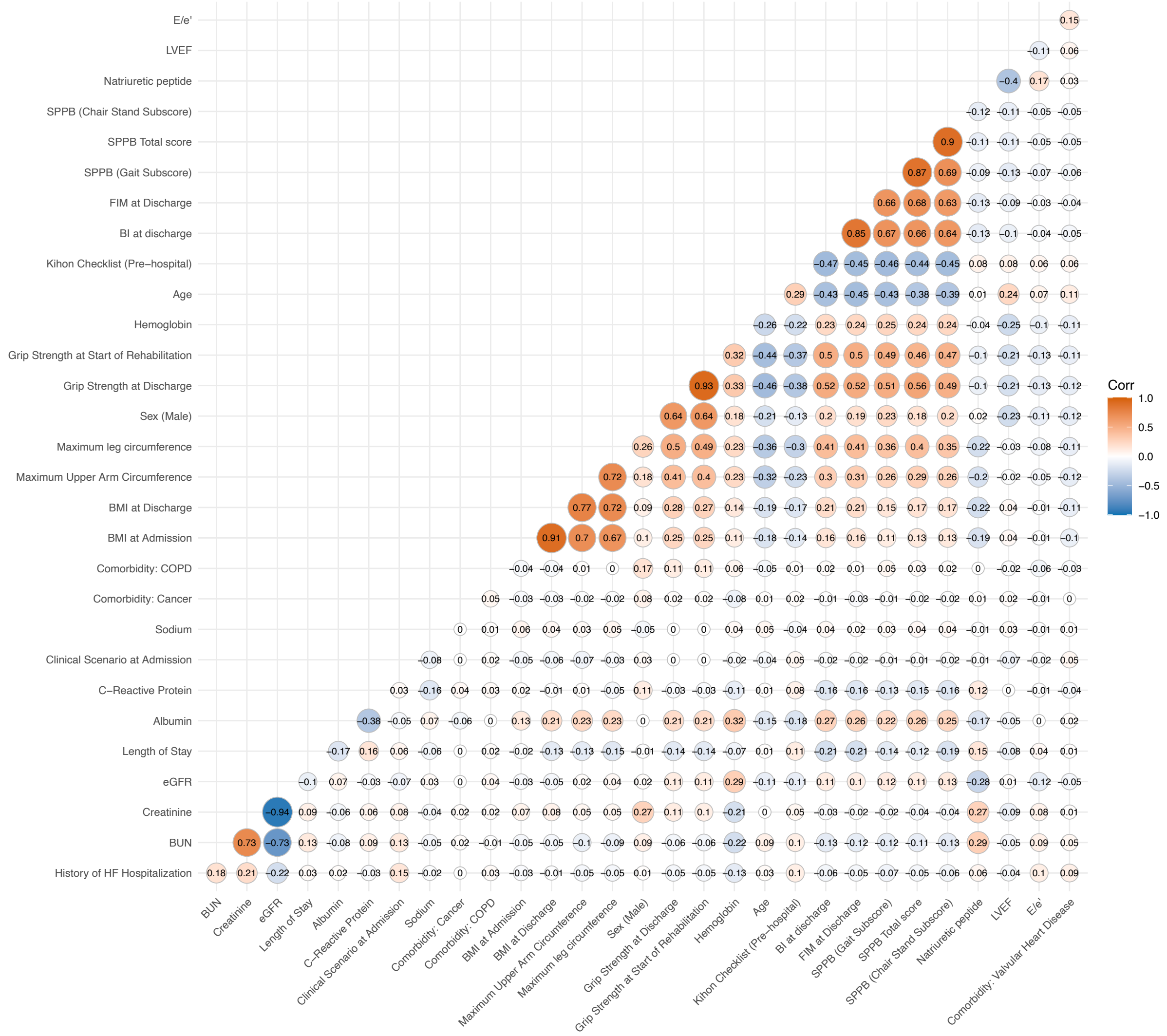

Supplementary Figure S6. Correlation matrix of the top predictor variables

Footnote: The heatmap shows the Spearman correlation coefficients between the top 30 predictor variables ranked by their mean absolute SHAP values. Variables were reordered using hierarchical clustering to group features with similar correlation patterns. Blue circles represent negative correlations and red circles represent positive correlations. Circle size and color intensity correspond to the absolute magnitude of the correlation coefficient. Only the lower triangle of the matrix is displayed, and correlation coefficients are printed within each circle.

Abbreviations: BI, Barthel Index; BMI, Body mass index; BUN, Blood Urea Nitrogen; COPD, Chronic Obstructive Pulmonary disease; E/e', ratio of early mitral inflow velocity to mitral annular early diastolic velocity; eGFR, estimated Glomerular Filtration Rate; FIM, Functional Independence Measure; HF, Heart Failure; LVEF, Left Ventricular Ejection Fraction; LOSO, leave-one-site-out; SHAP, SHapley Additive exPlanations; SPPB, Short Physical Performance Battery; XGBoost, eXtreme Gradient Boosting.

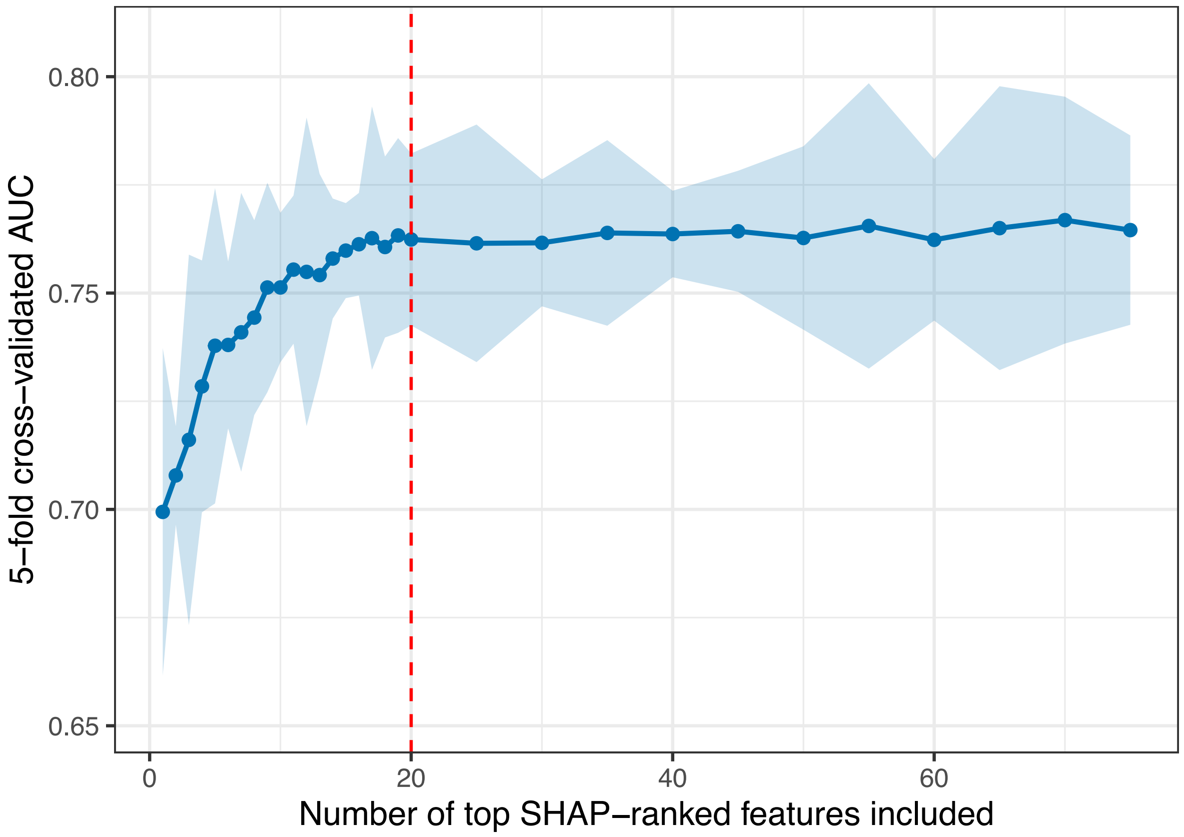

Supplementary Figure S7. Selection of the parsimonious model

Footnote: Feature ranking was based on mean absolute SHAP values obtained from the LOSO analyses. The plot shows the results of 5-fold cross-validation performed on the full analytical cohort while sequentially adding predictors in SHAP-ranked order. Model discrimination reached a plateau at approximately 20 predictors, marked by the dashed vertical line. The XGBoost 20-predictor model was chosen through clinical consensus rather than SHAP ranking alone. Because all models in this analysis were refitted using fixed hyperparameters, the cross-validated AUC values shown here do not necessarily match those of the full or parsimonious models.

Abbreviations: AUC, Area Under the Receiver Operating Characteristic Curve; LOSO, leave-one-site-out; SHAP, SHapley Additive exPlanations.

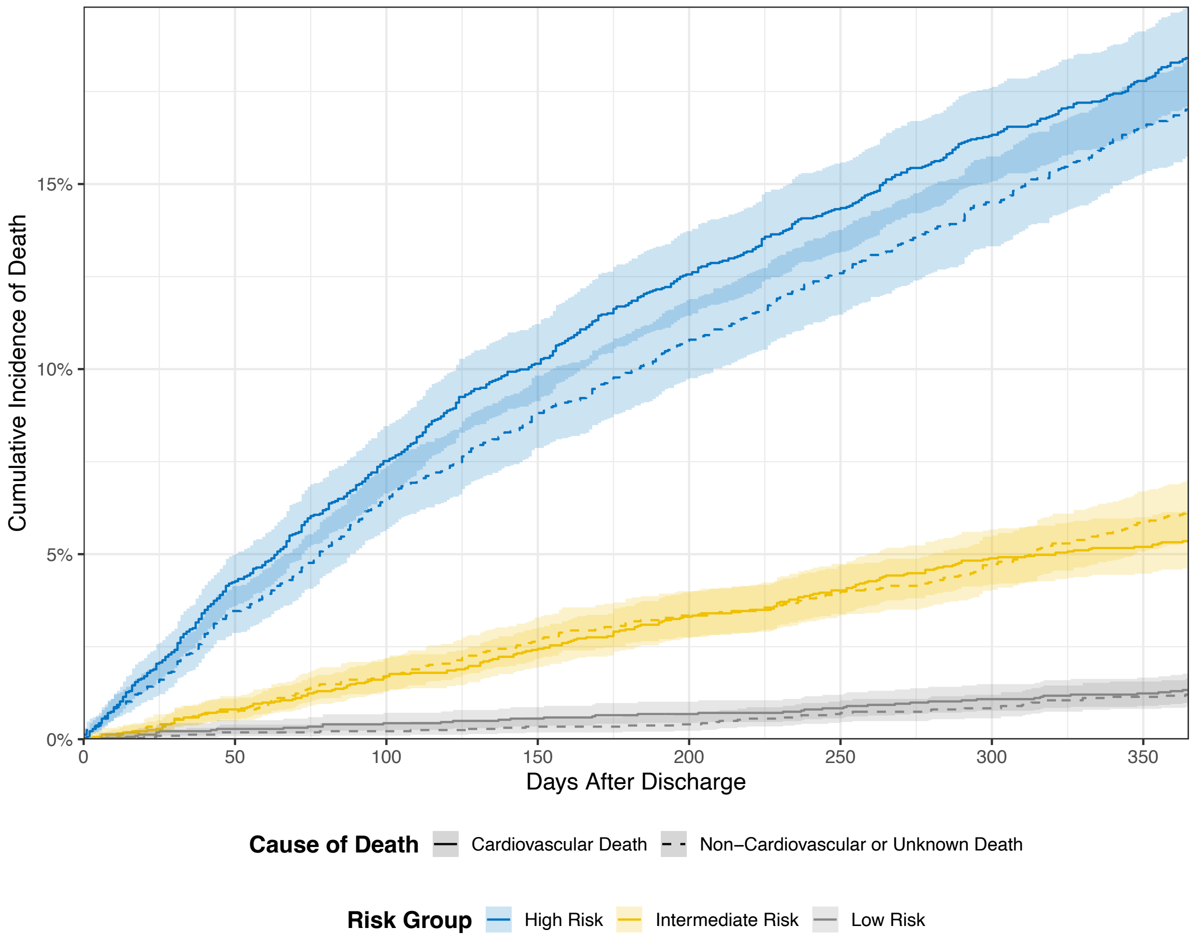

Supplementary Figure S8. Cumulative incidence of death by predicted risk group and cause of death

Footnote: The figure presents the cumulative incidence of death within one year after discharge, stratified by the three predicted risk groups derived from the final XGBoost model. Solid lines indicate cardiovascular death, and dashed lines indicate non-cardiovascular or unknown causes. Shaded areas represent 95% CI. High-risk patients had markedly higher cumulative incidence of both cardiovascular and non-cardiovascular mortality compared with the intermediate- and low-risk groups. Among cardiovascular deaths, the majority were attributed to worsening heart failure.

Abbreviations: CI, confidence interval; XGBoost, eXtreme Gradient Boosting.

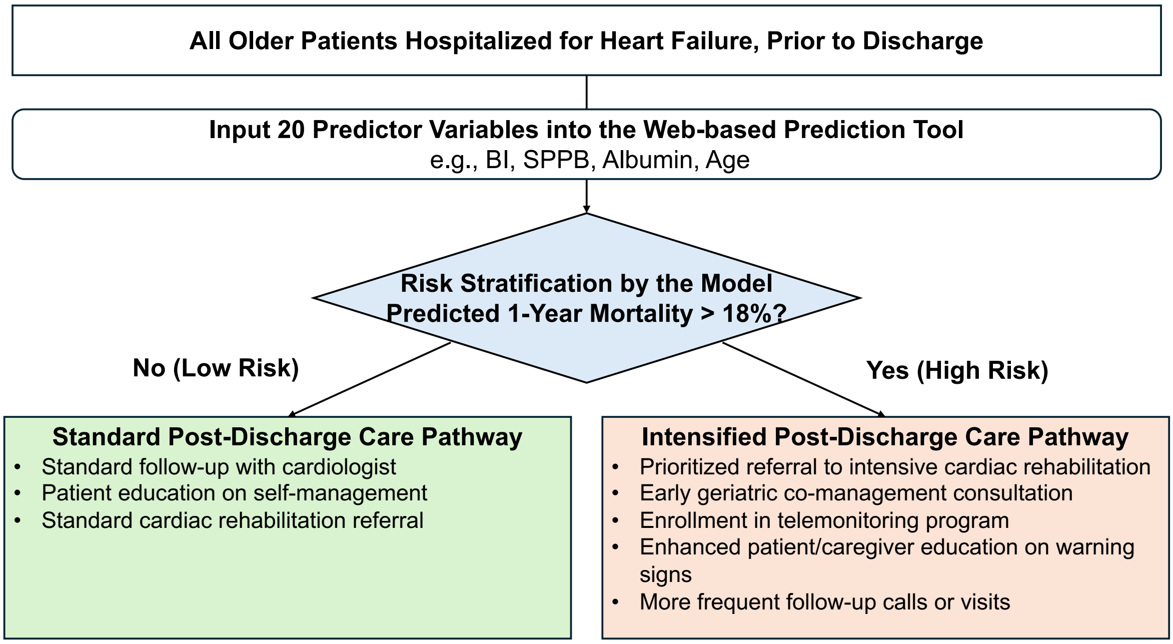

Supplementary Figure S9. Proposed clinical pathway for implementing the machine learning model in practice

Footnote: The diagram illustrates a potential workflow for using the web-based prediction tool to stratify older patients with heart failure at the time of hospital discharge. Based on the model's predicted 1-year mortality risk, patients are triaged into either a standard or an intensified post-discharge care pathway, enabling targeted allocation of healthcare resources to high-risk individuals. The risk threshold of 18% corresponds to the optimal cut-point determined by maximizing the Youden's index.

Abbreviations: BI, Barthel Index; SPPB, Short physical performance battery.

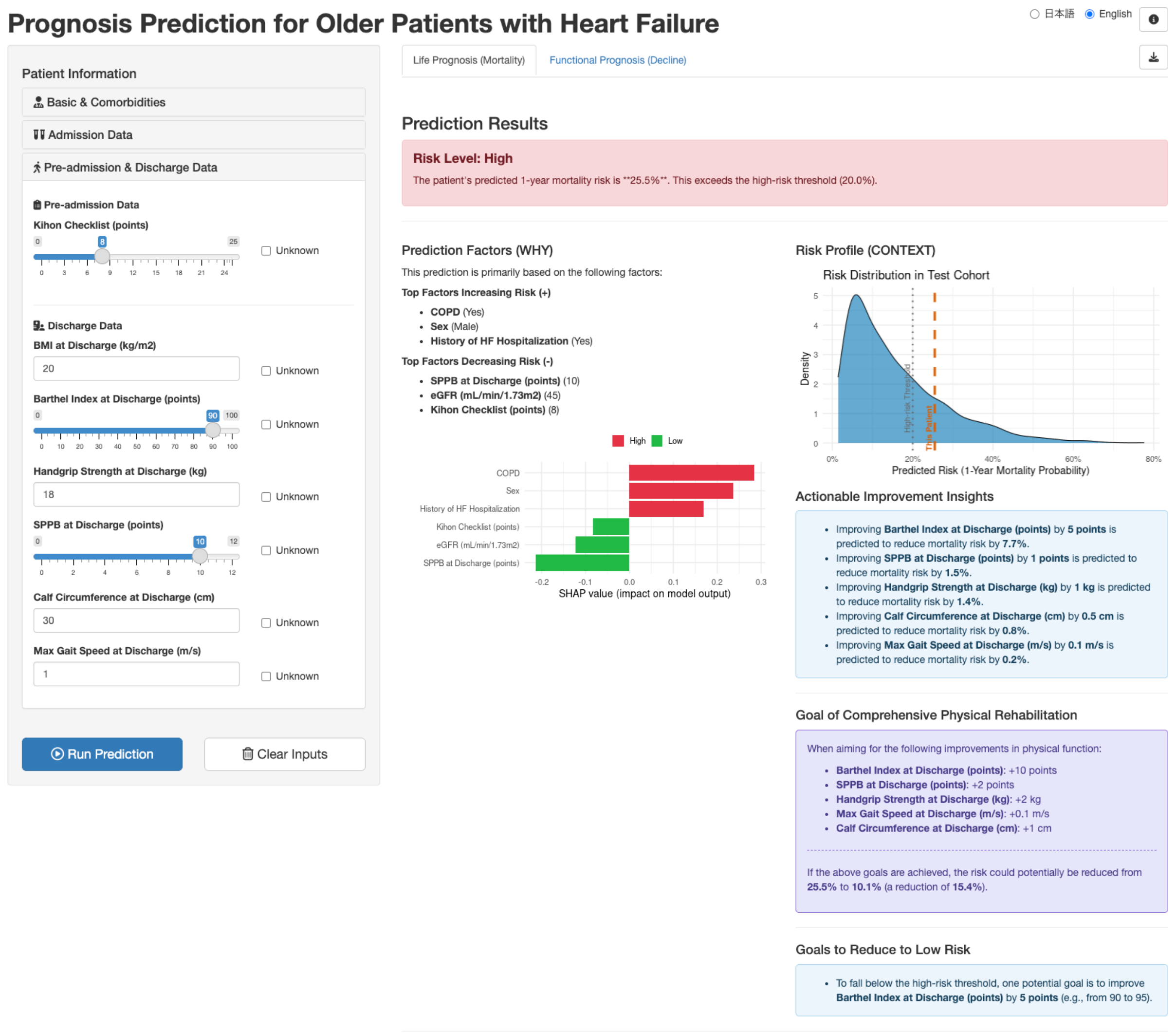

Supplementary Figure S10. Illustrative Workflow and User Interface of the Web-Based Prediction Tool

Footnote: The figure displays a representative screenshot from the prototype R Shiny web application developed to facilitate the clinical use of the Top-20 XGBoost model. The interface comprises several key sections:

Data Input (Left Panel): Clinicians can enter the 20 required predictor variables for an individual patient.

Prediction Output (Top Right): The tool returns the patient’s individualized 1-year mortality probability and corresponding risk category (e.g., "High Risk"). The patient's risk is also contextualized within the distribution of risks observed in the study cohort.

Prediction Explanation (Middle Right): An individualized SHAP-based plot visualizes the specific factors contributing to the patient's predicted risk, identifying which variables increased or decreased the estimate.

Actionable Insights (Bottom Right): The application simulates potential risk reduction based on improvements in modifiable functional parameters, supporting goal-setting for rehabilitation.
This tool is designed as a decision-support aid to enhance risk communication and post-discharge care planning. The application is currently a prototype, and its code is available from the corresponding author upon reasonable request.
Abbreviations: SHAP, SHapley Additive exPlanations; XGBoost, eXtreme Gradient Boosting.
